# A Software Tool for Calculating the Uncertainty of Diagnostic Accuracy Measures

**DOI:** 10.3390/diagnostics11030406

**Published:** 2021-02-27

**Authors:** Theodora Chatzimichail, Aristides T. Hatjimihail

**Affiliations:** Hellenic Complex Systems Laboratory, Kostis Palamas 21, 66131 Drama, Greece; tc@hcsl.com

**Keywords:** diagnostic accuracy measures, uncertainty, measurement uncertainty, sampling uncertainty, confidence intervals, diagnostic tests, screening tests

## Abstract

Screening and diagnostic tests are applied for the classification of people into diseased and non-diseased populations. Although diagnostic accuracy measures are used to evaluate the correctness of classification in clinical research and practice, there has been limited research on their uncertainty. The objective for this work was to develop a tool for calculating the uncertainty of diagnostic accuracy measures, as diagnostic accuracy is fundamental to clinical decision-making. For this reason, the freely available interactive program *Diagnostic Uncertainty* has been developed in the Wolfram Language. The program provides six modules with nine submodules for calculating and plotting the standard combined, measurement and sampling uncertainty and the resultant confidence intervals of various diagnostic accuracy measures of screening or diagnostic tests, which measure a normally distributed measurand, applied at a single point in time to samples of non-diseased and diseased populations. This is done for differing sample sizes, mean and standard deviation of the measurand, diagnostic threshold and standard measurement uncertainty of the test. The application of the program is demonstrated with an illustrative example of glucose measurements in samples of diabetic and non-diabetic populations, that shows the calculation of the uncertainty of diagnostic accuracy measures. The presented interactive program is user-friendly and can be used as a flexible educational and research tool in medical decision-making, to calculate and explore the uncertainty of diagnostic accuracy measures.

## 1. Introduction

**Figure 1:**
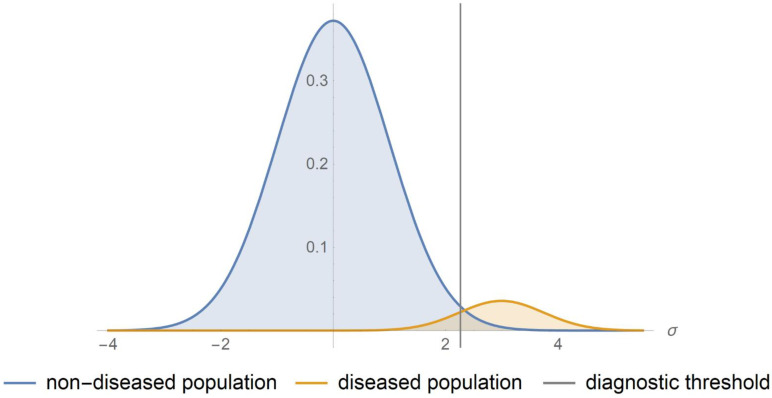
Probability density function plots. The probability density functions plots of a measurand in a non-diseased and diseased population.

Diagnosis in medicine is the determination of the nature of a disease condition [1]. The term diagnosis is derived from the Greek word “διάγνωσις” meaning “discernment”. It is assumed that there is a dichotomy between the populations with and without a disease condition. Diagnostic tests or procedures are applied for the classification of people into the respective disjoint groups. The probability distributions of the measurand of a quantitative diagnostic test in each of the diseased and non-diseased populations are overlapping. The results of a test though can be dichotomized, by assigning a diagnostic threshold or cutoff point (Figure 1) [1]. The possible test results are summarized in Table 1. It is assumed that there is a reference (“gold standard”) diagnostic method correctly classifying a subject as diseased or non-diseased [2]. The ratio of the diseased to the total population (diseased and non-diseased) at a single point in time is the prevalence rate (*r*) of the disease.

There is a persistent need of estimating the uncertainty of diagnostic accuracy measures, especially regarding screening and diagnostic tests of life-threatening diseases. The current pandemic of novel corona virus disease 2019 (COVID-19) has exposed this unequivocally [3,4,5,6,7]. There has been extensive research on either diagnostic accuracy or uncertainty, however, extremely limited research has been done on both subjects [8,9,10,11].

The program *Diagnostic Uncertainty* has been developed to explore the combined, measurement and sampling uncertainty of diagnostic accuracy measures as:

1. Diagnostic accuracy is fundamental to clinical decision-making [12],
2. Defining the permissible measurement uncertainty is critical to quality and risk management in laboratory medicine [13].
3. Sampling uncertainty is decisive for clinical study design to evaluate a screening or diagnostic test [14].

### 1.1. Diagnostic Accuracy Measures

There are diagnostic accuracy measures (DAM) used for evaluating the discriminative ability of a screening or diagnostic test in clinical research and practice [2]. These are [15]:

1. Error-based measures, estimating misclassification rates. These include sensitivity (*Se*), specificity (*Sp*), overall diagnostic accuracy (*ODA*), Youden’s index (*J*), Euclidean distance (*ED*) and concordance probability (*CZ*).
2. Information-based measures, assisting the interpretation of each single test result. These include positive predictive value (*PPV*), negative predictive value (*NPV*), likelihood ratio for positive result (*LR*+) and likelihood ratio for negative result (*LR*−).
3. Association-based measures, estimating the strength of the association between the test results and the reference diagnostic method. These include diagnostic odds ratio (*DOR*).

They can be further classified as following:

1. Defined conditionally on
  - The true disease condition status: sensitivity, specificity, overall diagnostic accuracy, diagnostic odds ratio, likelihood ratio for positive result, likelihood ratio for negative result, Youden’s index, Euclidean distance and concordance probability.
  - The test outcome: positive predictive value and negative predictive value.
2. As prevalence
  - Invariant: sensitivity, specificity, diagnostic odds ratio, likelihood ratio for positive result, likelihood ratio for negative result, Youden’s index, Euclidean distance and concordance probability.
  - Dependent: positive predictive value, negative predictive value and overall diagnostic accuracy.

The natural frequency and the probability definitions of the above diagnostic accuracy measures are presented in Table 2. The symbols are explained in Appendix A.

### 1.2. Uncertainty of Diagnostic Accuracy Measures

Uncertainty is an expression of imperfect or deficient information. When quantifiable it can be represented with probability [16]. The following components of the combined uncertainty of the diagnostic accuracy measures will be considered:

#### 1.2.1. Measurement Uncertainty

As measurements are inherently variable, measurement uncertainty is defined as a “parameter, associated with the result of a measurement, that characterizes the dispersion of the values that could reasonably be attributed to the measurand” [17]. Measurement uncertainty is replacing the total analytical error concept [18].

#### 1.2.2. Sampling Uncertainty

Diagnostic accuracy measures are estimated by applying a screening or diagnostic test to samples of populations. Sampling heterogeneity contributes to the combined uncertainty of the diagnostic accuracy measures [19]. Even when simple random sampling is applied, there is inherent sample heterogeneity [20]. A sample of size n is considered as simple random sample, if all possible samples of the same size are equally probable [21].

## 2. Materials and Methods

### 2.1. Computational Methods

For the calculation of the uncertainty of the diagnostic accuracy measures of a screening or diagnostic test based on a measurand, it is assumed that:

1. There is a reference (“gold standard”) diagnostic method classifying correctly a subject as diseased or non-diseased [22].
2. Either the values of the measurand or their transforms [23,24] are normally distributed in each of the diseased and non-diseased populations.
3. Measurement uncertainty is normally distributed and homoscedastic in the diagnostic threshold’s range.
4. The sampling is simple random.
5. If the measurement is above the threshold the patient is classified as test-positive, otherwise as test-negative.

#### 2.1.1. Calculation of Diagnostic Accuracy Measures

The calculation of the diagnostic accuracy measures is based on their probability definitions (Table 2). The sensitivity and specificity can be defined in terms of the error function and the complementary error function (see Appendix B). The other diagnostic accuracy measures can be expressed in terms of sensitivity, specificity and prevalence rate and calculated as shown in Appendix B.

#### 2.1.2. Calculation of Uncertainty of Diagnostic Accuracy Measures

The uncertainty of an input parameter or a diagnostic accuracy measure x can be expressed in the forms of standard and expanded uncertainty. The former, denoted as u(x) equals the standard deviation of x . The later, denoted as U(x) , is defined as an interval around x including x with probability p [25].

##### Measurement Uncertainty

The standard measurement uncertainty $u_{m}$ of a measurand is estimated from a sample of $n_{u}$ measurements, as described in “Guide to the expression of uncertainty in measurement”(GUM) and “Expression of measurement uncertainty in laboratory medicine” [17]. Bias may be considered as a component of the standard measurement uncertainty [26].

##### Sampling Uncertainty of Means and Standard Deviations

If $m_{P}$ and $s_{P}$ the mean and standard deviation of a measurand in a population sample of size $n_{P}$ , then the standard sampling standard uncertainties of $m_{P}$ and $s_{P}$ are:

(1) $$u_{s}\left(m_{P}\right)=\frac{s_{P}}{\sqrt{n_{P}}}$$

(2) $$u_{s}\left(s_{P}\right)=\frac{s_{P}}{\sqrt{2\left(n_{P}-1\right)}}$$

##### Combined Uncertainty of Means and Standard Deviations

If $u_{m}$ the standard measurement uncertainty of a screening or diagnostic test measuring a measurand and $m_{P}$ and $s_{P}$ the mean and standard deviation of the measurand in a population sample of size $n_{P}$ , then the standard combined uncertainties of the mean $m_{P}$ and standard deviation $s_{P}$ are:

(3) $$u_{c}\left(m_{P}\right)=\sqrt{\frac{s_{P}^{2}}{n_{P}}+u_{m}^{2}}$$

(4) $$u_{c}\left(s_{P}\right)=\sqrt{\frac{s_{P}^{2}}{2\left(n_{P}-1\right)}+u_{m}^{2}}$$

##### Sampling Uncertainty of Prevalence Rate

If $n_{\bar{D}}$ and $n_{D}$ the respective numbers of non-diseased and diseased in a population sample, then the standard uncertainty of the prevalence rate $r=\frac{n_{D}}{n_{\bar{D}}+n_{D}}$ of the disease can be approximated as:

(5) $$u_{s}\left(r\right)=\sqrt{\frac{\left(2+n_{\bar{D}})(2+n_{D}\right)}{{\left(4+n_{\bar{D}}+n_{D}\right)}^{3}}}$$

according to the Agresti–Coull adjustment of the Waldo interval [27].

##### Combined Uncertainty of Diagnostic Accuracy Measures

The standard combined uncertainty $u_{c}\left(x\right)$ of each diagnostic accuracy measure x is calculated by applying the rules of uncertainty propagation from the input values to the calculated diagnostic accuracy measure (see Appendix B), according to GUM [28,29], with a first-order Taylor series approximation to uncertainty propagation [30].

When there are l components of uncertainty, with standard uncertainties $u_{i}\left(x\right)$ respectively, then:

(6) $$u_{c}\left(x\right)=\sqrt{\sum_{i=1}^{l}u_{i}{\left(x\right)}^{2}}$$

##### Expanded Uncertainty of Diagnostic Accuracy Measures

The effective degrees of freedom $v_{eff}$ of the standard combined uncertainty $u_{c}\left(x\right)$ are calculated using the Welch–Satterthwaite formula [31,32]:

(7) $$v_{eff}=\frac{u_{c}{\left(x\right)}^{4}}{\sum_{i=1}^{l}\frac{u_{i}{\left(x\right)}^{4}}{v_{i}}}$$

If $v_{min}$ the minimum of the respective degrees of freedom $v_{1},\;v_{2},\ldots ,v_{l}$ then:

(8) $$v_{min}\leq v_{eff}\leq \overset{l}{\underset{i=1}{\sum }}v_{i}\;$$

If $F_{v}\left(z\right)$ the cumulative distribution function of the Student’s *t*-distribution with v degrees of freedom and $u_{c}\left(x\right)\;$ the standard combined uncertainty of a diagnostic accuracy measure x, its expanded combined uncertainty, at a confidence level p , is calculated as:

(9) $$U_{c}\left(x\right)=\left(F_{v}^{-1}\left(\frac{1-p}{2}\right)u_{c}\left(x\right),F_{v}^{-1}\left(\frac{1+p}{2}\right)u_{c}\left(x\right)\right)$$

The resultant confidence interval (CI) of x , at the same confidence level p , is:

(10) $$CI_{p}\left(x\right)=\left(x+F_{v}^{-1}\left(\frac{1-p}{2}\right)u_{c}\left(x\right),x+F_{v}^{-1}\left(\frac{1+p}{2}\right)u_{c}\left(x\right)\right)$$

### 2.2. The Program

To calculate the uncertainty of the diagnostic accuracy measures, the interactive program *Diagnostic Uncertainty* was developed in the Wolfram Language [33], using Wolfram Mathematica^®^ Ver. 12.2, Wolfram Research, Inc., Champaign, IL, USA [34]. The program was designed to provide six modules with nine submodules, for calculating and plotting the standard combined, measurement and sampling uncertainty and the resultant confidence intervals of various diagnostic accuracy measures of a screening or diagnostic test, applied at a single point in time in non-diseased and diseased population samples. The test measures a measurand in the population samples, for varying values of their sizes, mean and standard deviation and standard measurement uncertainty of the measurand. It is assumed that the measurands and measurement uncertainty are normally distributed and that measurement uncertainty is homoscedastic.

The program is freely available as a Wolfram Mathematica Notebook (.nb) (Supplementary File: Uncertainty.nb). It can be run on Wolfram Player^®^ or Wolfram Mathematica^®^ (see Appendix C).

## 3. Results

### 3.1. Flowchart of the Program

**Figure 2:**
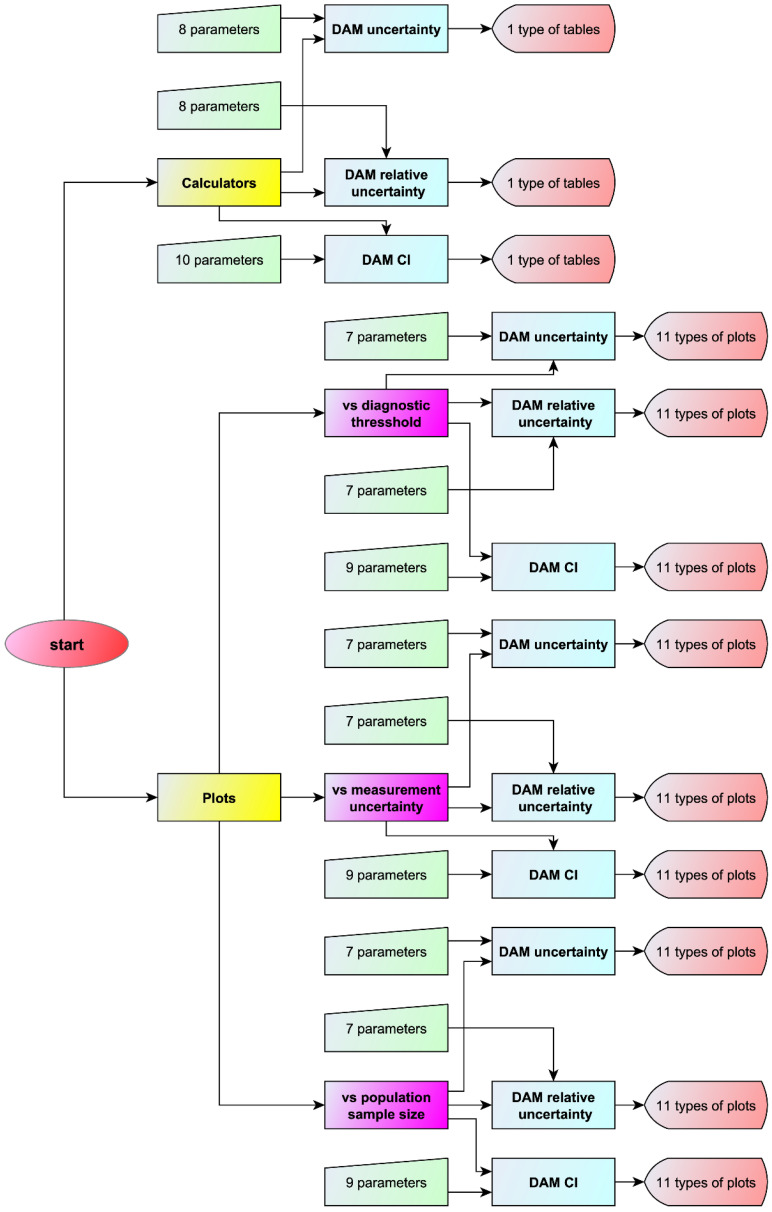
Program flowchart. The flowchart of the program with the number of the input parameters and of the output types for each module or submodule.

The flowchart of the program is presented in Figure 2.

### 3.2. Interface of the Program

The modules and submodules of the program include panels with controls which allow the interactive manipulation of various parameters, as described in detail in Supplementary File: Diagnostic Uncertainty Interface.pdf. These are the following:

#### 3.2.1. Plots vs. Diagnostic Threshold Module

##### Diagnostic Accuracy Measures Standard Uncertainty Plots Submodule

**Figure 3:**
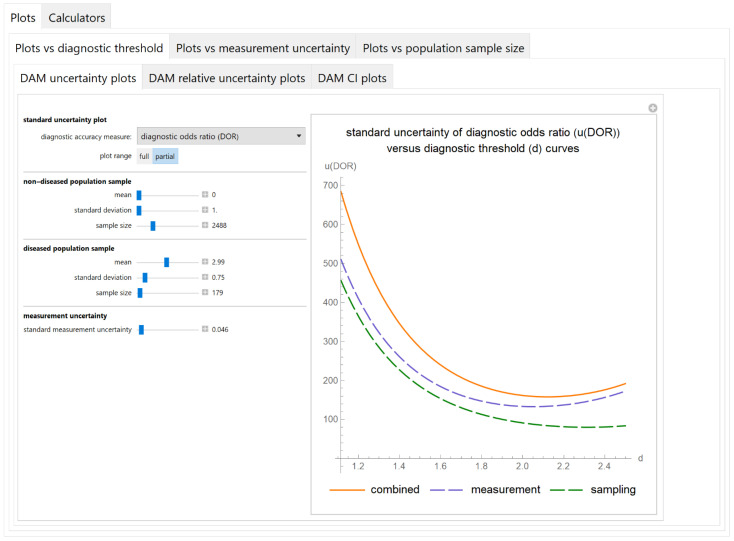
Plots vs. diagnostic threshold module, DAM uncertainty plots submodule screenshot. Standard combined, measurement and sampling uncertainty of diagnostic odds ratio *(u*(*DOR*)) versus diagnostic threshold (*d*) curve plot, with the settings shown on the left. The respective parameter settings are also shown in Table 3.

The values of the standard combined, measurement and sampling uncertainties of diagnostic accuracy measures of a screening or diagnostic test are plotted versus the diagnostic threshold of the test (Figure 3).

##### Diagnostic Accuracy Measures Relative Standard Uncertainty Plots Submodule

**Figure 4:**
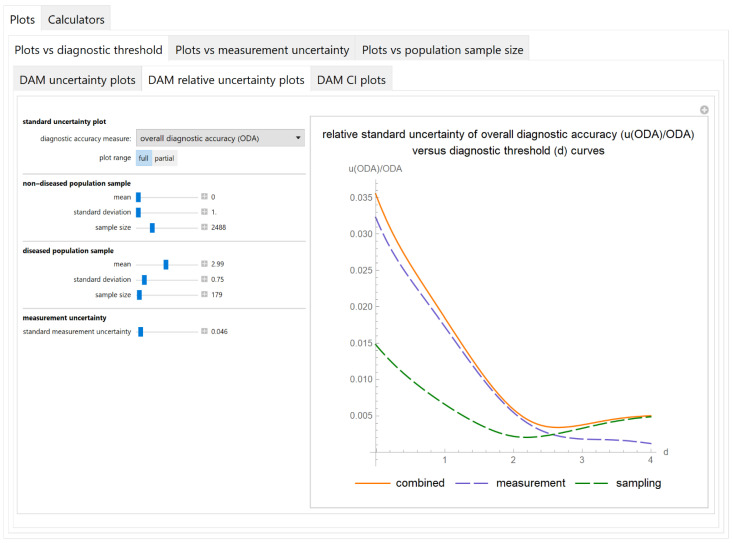
Plots vs. diagnostic threshold module, DAM relative uncertainty plots submodule screenshot. Relative standard combined, measurement and sampling uncertainty of overall diagnostic accuracy (*u*(*ODA*)/*ODA*) versus diagnostic threshold (*d*) curve plot, with the settings shown on the left. The respective parameter settings are also shown in Table 3.

The values of the relative standard combined, measurement and sampling uncertainties of diagnostic accuracy measures of a screening or diagnostic test are plotted versus the diagnostic threshold of the test (Figure 4).

##### Confidence Intervals of Diagnostic Accuracy Measures Plots Submodule

**Figure 5:**
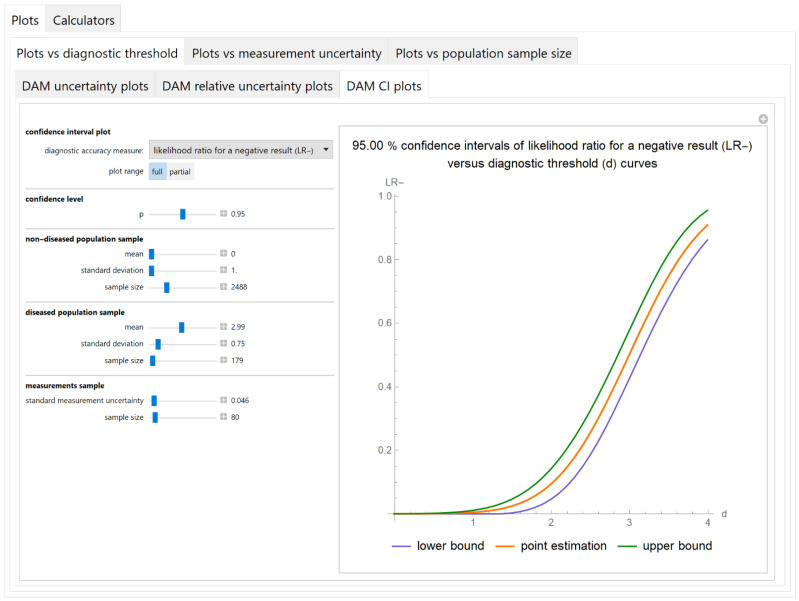
Plots vs. diagnostic threshold module, DAM CI plots submodule screenshot. Confidence intervals of likelihood ratio for a negative test result (*LR*−) versus diagnostic threshold (*d*) curves plot, with the settings shown on the left. The respective parameter settings are also shown in Table 3.

The values of the lower and upper bounds of the confidence intervals of diagnostic accuracy measure of a screening or diagnostic test, at a selected confidence level, are plotted versus the diagnostic threshold of the test (Figure 5).

#### 3.2.2. Plots vs. Measurement Uncertainty Module

##### Diagnostic Accuracy Measures Standard Uncertainty Plots Submodule

**Figure 6:**
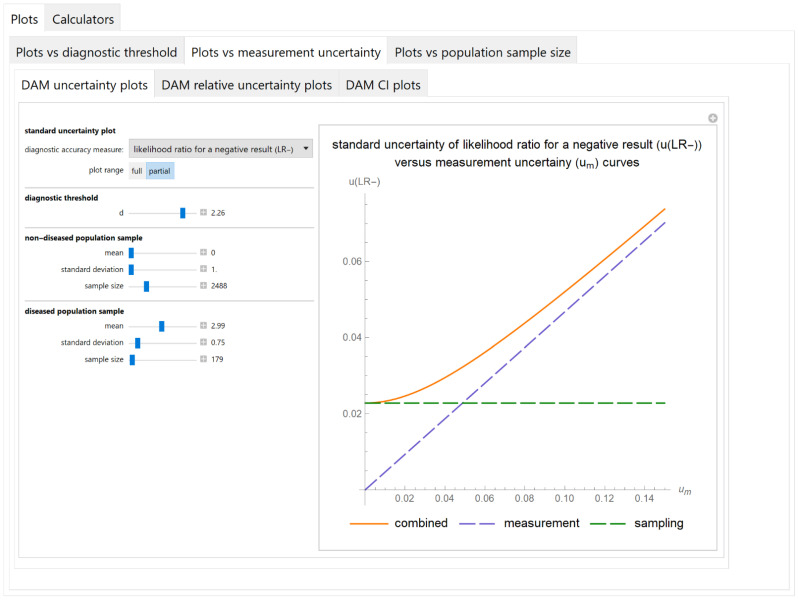
Plots vs. measurement uncertainty module, DAM uncertainty plots submodule screenshot. Standard combined, measurement and sampling uncertainty of likelihood ratio for a negative test result (*u*(*LR*−)) versus standard measurement uncertainty (*u_m_*) curve plot, with the settings shown on the left. The respective parameter settings are also shown in Table 3.

The values of the standard combined, measurement and sampling uncertainties of diagnostic accuracy measures of a screening or diagnostic test are plotted versus the measurement uncertainty of the test (Figure 6).

##### Diagnostic Accuracy Measures Relative Standard Uncertainty Plots Submodule

**Figure 7:**
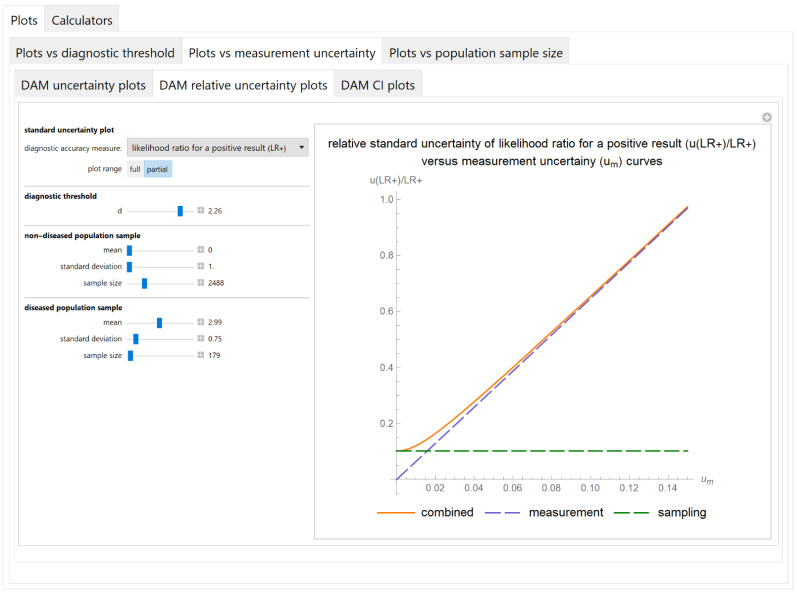
Plots vs. measurement uncertainty module, DAM relative uncertainty plots submodule screenshot. Relative standard combined, measurement and sampling uncertainty of likelihood ratio for a positive test result (*u*(*LR*+)/*LR*+) versus measurement uncertainty (*u_m_*) curves plot, with the settings shown on the left. The respective parameter settings are also shown in Table 3.

The values of the relative standard combined, measurement and sampling uncertainties of diagnostic accuracy measures of a screening or diagnostic test are plotted versus the measurement uncertainty of the test (Figure 7).

##### Confidence Intervals of Diagnostic Accuracy Measures Plots Submodule

**Figure 8:**
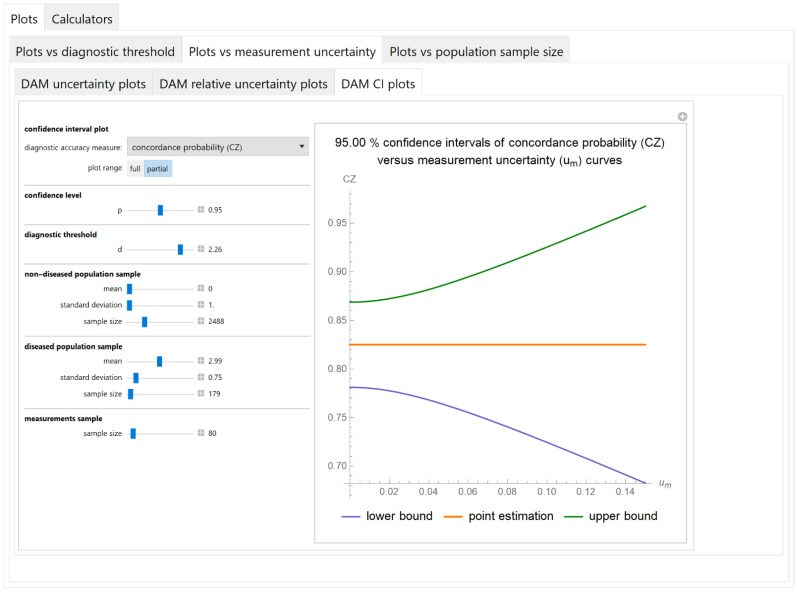
Plots vs. measurement uncertainty module, DAM CI plots submodule screenshot. Confidence intervals of concordance probability (*CZ*) versus standard measurement uncertainty (*u_m_*) curves plot, with the settings shown on the left. The respective parameter settings are also shown in Table 3.

The values of the lower and upper bounds of the confidence intervals of diagnostic accuracy measures of a screening or diagnostic test, at a selected confidence level, are plotted versus the measurement uncertainty of the test (Figure 8).

#### 3.2.3. Plots vs. Population Sample Size Module

##### Diagnostic Accuracy Measures Standard Uncertainty Plots Submodule

**Figure 9:**
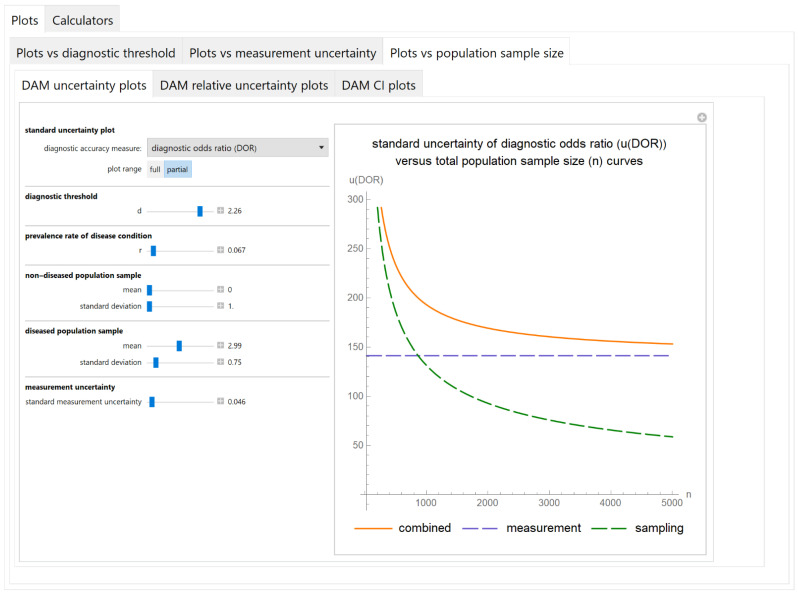
Plots vs. population sample size module, DAM uncertainty plots submodule screenshot. Standard combined, measurement and sampling uncertainty of diagnostic odds ratio (*u*(*DOR*)) versus total population sample size (*n*) curves plot, with the settings shown on the left. The respective parameter settings are also shown in Table 3.

The values of the standard combined, measurement and sampling uncertainties of diagnostic accuracy measures of a screening or diagnostic test are plotted versus the total population sample size (Figure 9).

##### Diagnostic Accuracy Measures Relative Standard Uncertainty Plots Submodule

**Figure 10:**
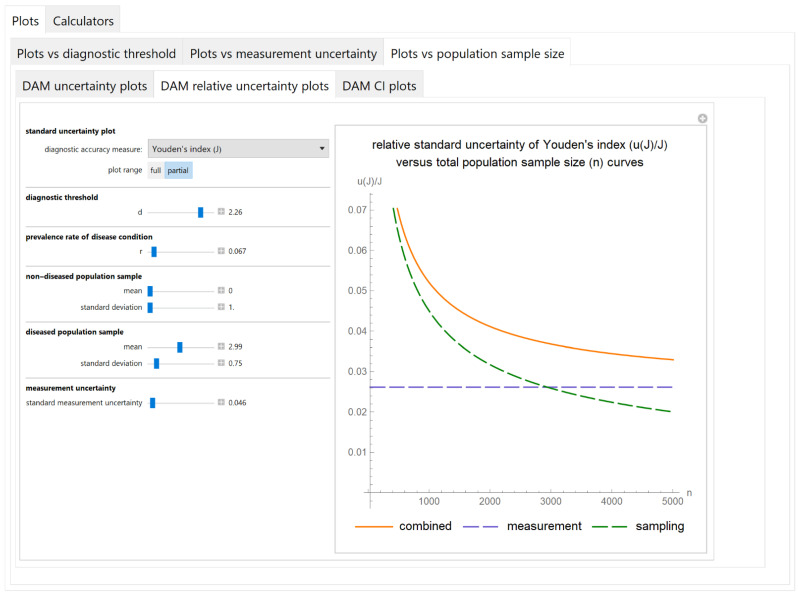
Plots vs. population sample size module, DAM relative uncertainty plots submodule screenshot. Relative standard combined, measurement and sampling uncertainty of Youden’s index (*u*(*J*)/*J*) versus total population sample size (*n*) curves plot, with the settings shown on the left. The respective parameter settings are also shown in Table 3.

The values of the relative standard combined, measurement and sampling uncertainties of diagnostic accuracy measures of a screening or diagnostic test are plotted versus the total population sample size (Figure 10).

##### Confidence Intervals of Diagnostic Accuracy Measures Plots Submodule

**Figure 11:**
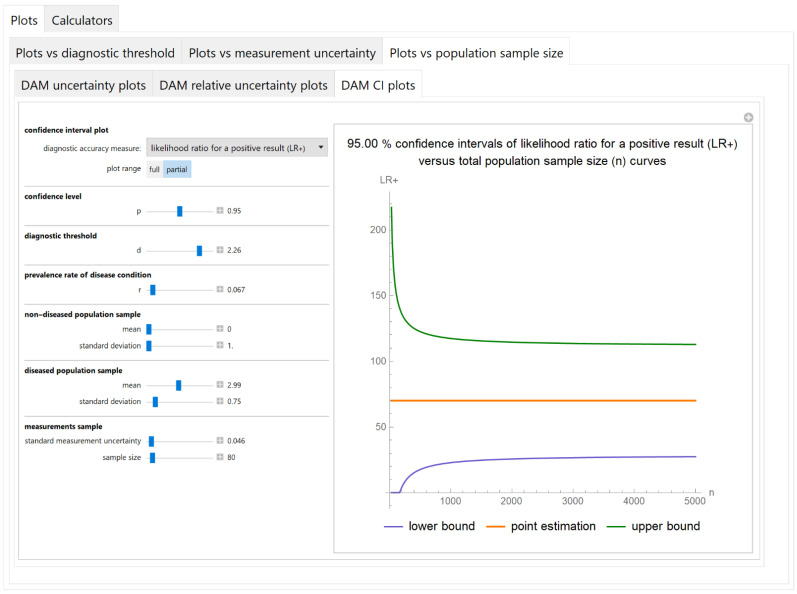
Plots vs. population sample size module, DAM CI plots submodule screenshot. Confidence intervals of likelihood ratio for a positive test result (*LR*+) versus total population sample size (*n*) curves plot, with the settings shown at the left. The respective parameter settings are also shown in Table 3.

The values of the lower and upper bounds of the confidence intervals of diagnostic accuracy measures of a screening or diagnostic test, at a selected confidence level, are plotted versus the total population sample size (Figure 11).

#### 3.2.4. Diagnostic Accuracy Measures Standard Uncertainty Calculator Module

**Figure 12:**
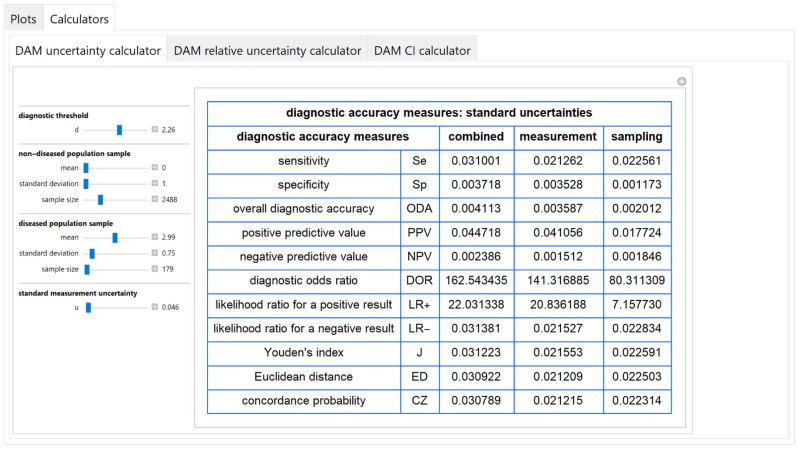
DAM uncertainty calculator module screenshot. Calculated standard combined, measurement and sampling uncertainties of diagnostic accuracy measures, with the settings shown on the left. The respective parameter settings are also shown in Table 3.

The values of the standard combined, measurement and sampling uncertainties of diagnostic accuracy measures of a screening or diagnostic test, at a selected diagnostic threshold, are calculated and presented in a table (Figure 12).

#### 3.2.5. Diagnostic Accuracy Measures Relative Standard Uncertainty Calculator Module

**Figure 13:**
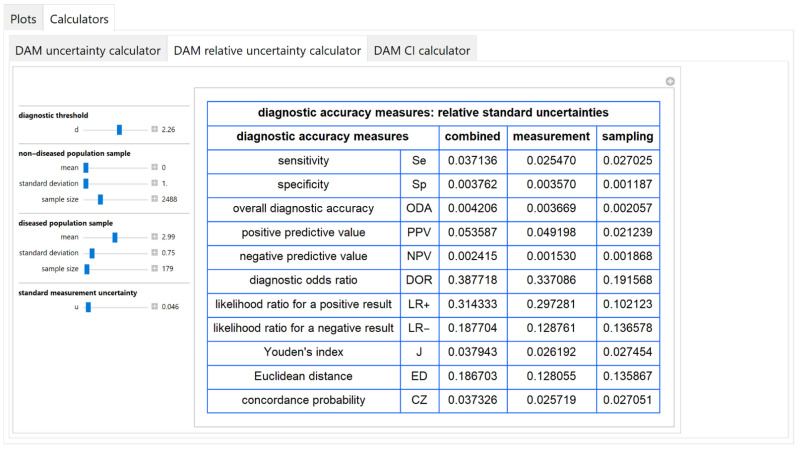
DAM relative uncertainty calculator submodule screenshot. Calculated relative standard combined, measurement and sampling uncertainty of diagnostic accuracy measures, with the settings shown on the left. The respective parameter settings are also shown in Table 3.

The values of the relative standard combined, measurement and sampling uncertainties of diagnostic accuracy measures of a screening or diagnostic test, at a selected diagnostic threshold, are calculated and presented in a table (Figure 13).

#### 3.2.6. Diagnostic Accuracy Measures Confidence Intervals Calculator Module

**Figure 14:**
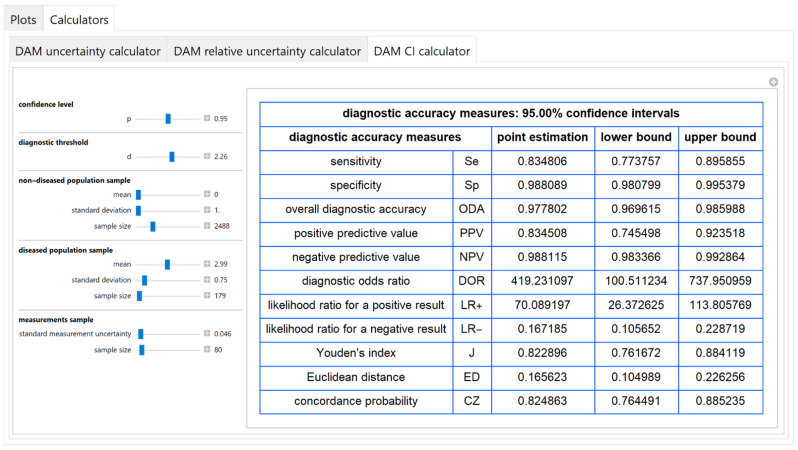
DAM CI calculator module screenshot. Calculated point estimations and confidence intervals of diagnostic accuracy measures, with the settings shown on the left. The respective parameter settings are also shown in Table 3.

The point estimations and the lower and upper bounds of the confidence intervals of diagnostic accuracy measures of a screening or diagnostic, at a selected confidence level and diagnostic threshold, are calculated and presented in a table (Figure 14).

### 3.3. Illustrative Example

The program was applied to a bimodal distribution of log-transformed blood glucose measurements in samples of non-diabetic and diabetic populations. The data were derived from a national health survey conducted in Malaysia in 1996 [35]. A glucose tolerance test (OGTT) was performed on 2667 Malay adults, aged 40–49 years. The respective sizes of the samples of the diseased and non-diseased populations were 179 and 2488. Glucose was measured with reflectance photometry, after the ingestion of 75 g glucose monohydrate. It was assumed that the measurement coefficient of variation and bias were equal to 4% and 2%, respectively. The log-transformed measurands of each population were normally distributed, as shown in Figure 1. The standardized log-transformed measurand means and standard deviations of the samples of the diseased and non-diseased populations, the standard measurement uncertainty and the diagnostic threshold were expressed in units equal to the standard deviation of the log-transformed measurand of the sample of the non-diseased population. The standardized log-transformed standard measurement uncertainty 0.046 of the test corresponds to coefficient of variation equal to 2%. The standardized log-transformed American Diabetes Association (ADA) diagnostic threshold for diabetes of the 2-h postprandial glucose during OGTT is equal to 2.26 [36].

The results of the illustrative example are presented:

1. In the plots of Figure 3, Figure 4, Figure 5, Figure 6, Figure 7, Figure 8, Figure 9, Figure 10 and Figure 11 and Figure 15, Figure 16, Figure 17, Figure 18, Figure 19, Figure 20 and Figure 21.
2. In the chart of Figure 22
3. In the tables of Figure 12, Figure 13 and Figure 14.

The parameter settings of Figure 3, Figure 4, Figure 5, Figure 6, Figure 7, Figure 8, Figure 9, Figure 10, Figure 11, Figure 12, Figure 13 and Figure 14 are presented in Table 3 and of Figure 15, Figure 16, Figure 17, Figure 18, Figure 19, Figure 20, Figure 21 and Figure 22 in Table 4. Figure 15, Figure 16, Figure 17, Figure 18, Figure 19, Figure 20 and Figure 21 present the standard combined, measurement and sampling uncertainty and the resultant confidence intervals of sensitivity, specificity, positive and negative predictive value versus diagnostic threshold, measurement uncertainty and total population sample size.

The combined uncertainty and the resultant confidence intervals increase with measurement uncertainty (Figure 6, Figure 7 and Figure 8, Figure 18 and Figure 19) and decrease with total population sample size (Figure 9, Figure 10 and Figure 11, Figure 20 and Figure 21).

**Figure 22:**
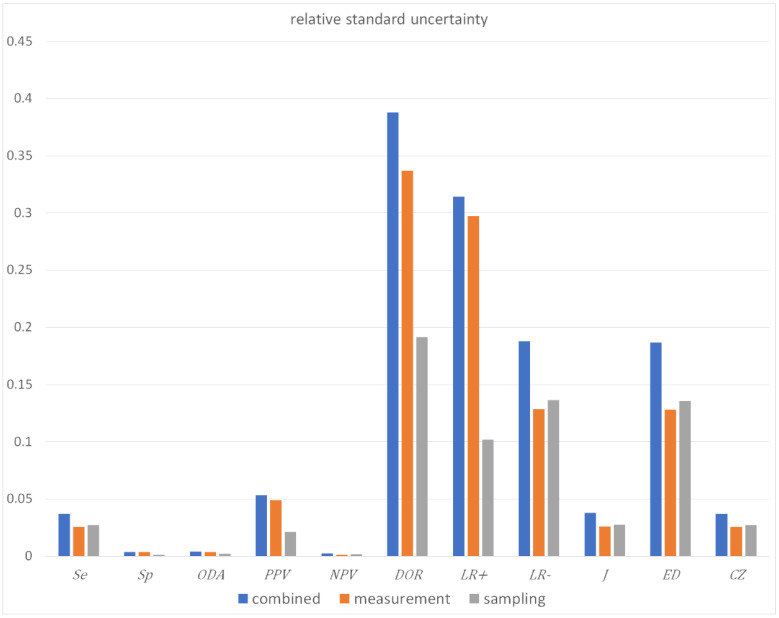
Histogram of standard combined, measurement and sampling uncertainties of diagnostic accuracy measures, with the respective parameter settings in Table 4.

In the illustrative example, combined uncertainty *u_c_*(*x*)has (see Figure 13 and Figure 22):

1. Little effect $\left(\left(u_{c}\left(x\right)/x\right)\;<\;0.5\%\right)$ on specificity, overall diagnostic accuracy and negative predictive value,
2. Intermediate effect $\left(3.5\%\;<\;\left(u_{c}\left(x\right)/x\right)\;<\;5.5\%\right)$ on sensitivity, positive predictive value, Youden’s index and concordance probability,
3. Greater effect $\left(18\%\;<\;\left(u_{c}\left(x\right)/x\right)\;<\;39\%\right)$ on diagnostic odds ratio, on likelihood ratio for a positive or negative result and Euclidean distance, in accordance with previous findings [37,38].

In addition, measurement uncertainty is the main component of the combined uncertainty of specificity, overall diagnostic accuracy, positive predictive value, diagnostic odds ratio and likelihood ratio for a positive result.

## 4. Discussion

The program *Diagnostic Uncertainty* explores the combined, measurement and sampling uncertainty of diagnostic accuracy measures of a screening or diagnostic test (Figure 3, Figure 4, Figure 6, Figure 7, Figure 9, Figure 10 and Figure 11, Figure 12 and Figure 13) and the resultant confidence intervals (Figure 5, Figure 8, Figure 11 and Figure 14). Combined uncertainty and the resultant confidence intervals depend on the diagnostic threshold (Figure 3, Figure 4 and Figure 5 and Figure 15, Figure 16 and Figure 17), on measurement uncertainty (Figure 6, Figure 7 and Figure 8, Figure 18 and Figure 19) and on population parameters, including the total population sample size (Figure 9, Figure 10 and Figure 11, Figure 20 and Figure 21).

The complexity of the calculations of the confidence intervals of the diagnostic accuracy measures is considerable. In antithesis of the complexity of the calculations, the program simplifies its exploration with a user-friendly interface. Furthermore, it provides calculators for the calculation of the components of uncertainty of diagnostic accuracy measures and the resultant confidence intervals (Figure 12, Figure 13 and Figure 14).

As demonstrated by the illustrative example described above, in this instance uncertainty has relatively little effect on specificity, overall diagnostic accuracy and negative predictive value. It affects more sensitivity, positive predictive value, Youden’s index and concordance probability, while it has a considerable impact on diagnostic odds ratio, likelihood ratio for a positive or negative result and Euclidean distance (Figure 22). However, further research is needed to explore the uncertainty of diagnostic accuracy measures with different clinically- and laboratory-relevant parameter settings.

Limitations of this program, that could be improved by further research, are the following:

(1) The assumptions used for the calculations:
  - The existence of a “gold standard” diagnostic method. If a “gold standard” does not exist, there are alternative approaches for the estimation of diagnostic accuracy measures [39].
  - The normality of either the measurements or their applicable transforms [23,24,40,41], however, this is usually valid. There is related literature on the distribution of measurements of diagnostic tests, in the context of reference intervals and diagnostic thresholds or clinical decision limits [42,43,44,45,46].
  - The bimodality of the measurands, that is generally accepted, although unimodal distributions could be considered [47,48].
  - The simple random sampling.
  - The measurement uncertainty homoscedasticity in the diagnostic thresholds range. Nevertheless, if measurement uncertainty is heteroscedastic, thus skewing the measurements distribution, appropriate transformations may restore homoscedasticity [49].

If the above assumptions are not valid, there are other components of uncertainty which are not calculated by this program.

(2) The first order Taylor series approximations for the uncertainty propagation calculations [28,30]. Higher order approximations may improve the accuracy.
(3) The uncertainty of prevalence rate approximation by the Agresti–Coull adjusted Waldo interval [25], although there are more exact methods [50].

However, addressing these limitations, would increase exponentially the computational complexity.

The program presented in this work complements our previously published software [11], which explores the effects of measurement uncertainty on diagnostic accuracy measures applied to populations. This program calculates the standard and expanded combined, measurement and sampling uncertainty and the resultant confidence intervals of diagnostic accuracy measures of diagnostic tests, applied to samples of populations, providing 99 different types of plots and three different types of comprehensive tables (Figure 2), many of which are novel. To the best of our knowledge, no software, including all major general or medical statistical and uncertainty related software packages (Matlab^®^, NCSS^®^, R, SAS^®^, SPSS^®^, Stata^®^, MedCalc^®^, NIST Uncertainty Machine, UQLab, metRology), provides this range of plots and tables without advanced programming.

## 5. Conclusions

The presented program *Diagnostic Uncertainty* calculates the combined, measurement and sampling uncertainty of diagnostic accuracy measures and the resultant confidence intervals and can be used as a flexible, user-friendly, interactive educational or research tool in medical decision-making.

## Figures and Tables

**Figure 15:**
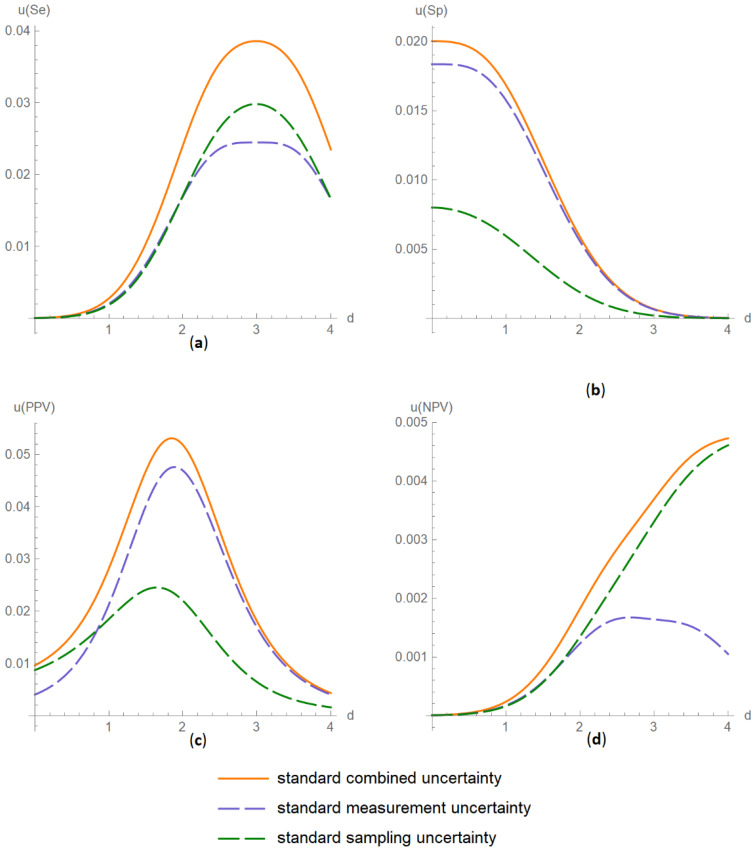
DAM standard uncertainties versus diagnostic threshold plots. Plots of standard combined, measurement and sampling uncertainties of (**a**) sensitivity (*u*(*Se*)), (**b**) specificity (*u*(*Sp*)), (**c**) positive predictive value (*u*(*PPV*)) and (**d**) negative predictive value (*u*(*NPV*)) versus diagnostic threshold (*d*) curves, with the respective parameter settings in Table 4.

**Figure 16:**
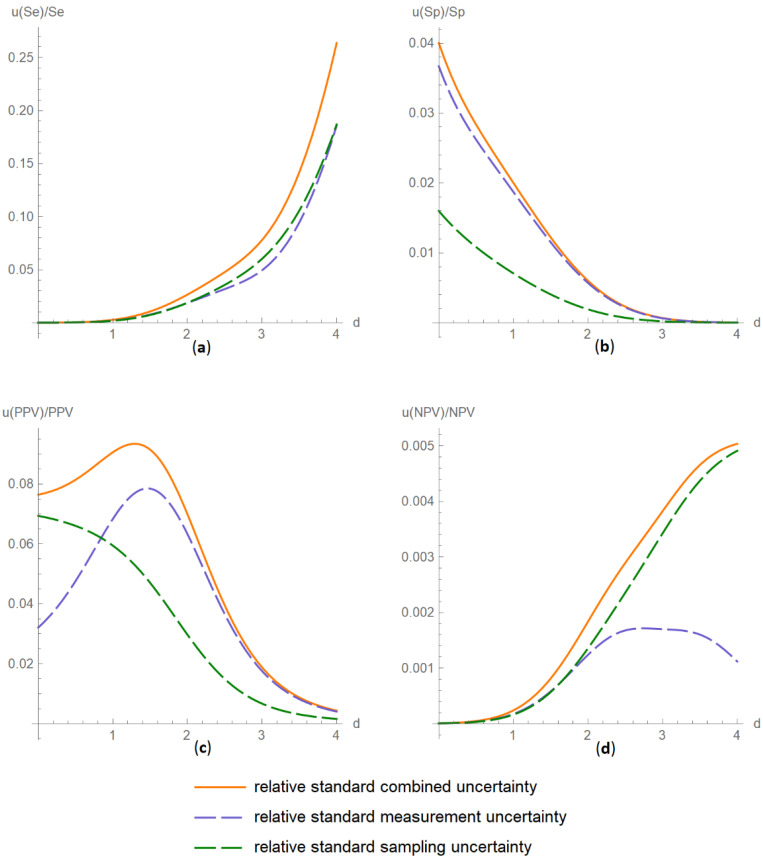
DAM relative standard uncertainties versus diagnostic threshold plots. Plots of relative standard combined, measurement and sampling uncertainties of (**a**) sensitivity (*u*(*Se*)/*Se*), (**b**) specificity (*u*(*Sp*)/*Sp*), (**c**) positive predictive value (*u*(*PPV*)/*PPV*) and (**d**) negative predictive value (*u*(*NPV*)/*NPV*) versus diagnostic threshold (*d*) curves, with the respective parameter settings in Table 4.

**Figure 17:**
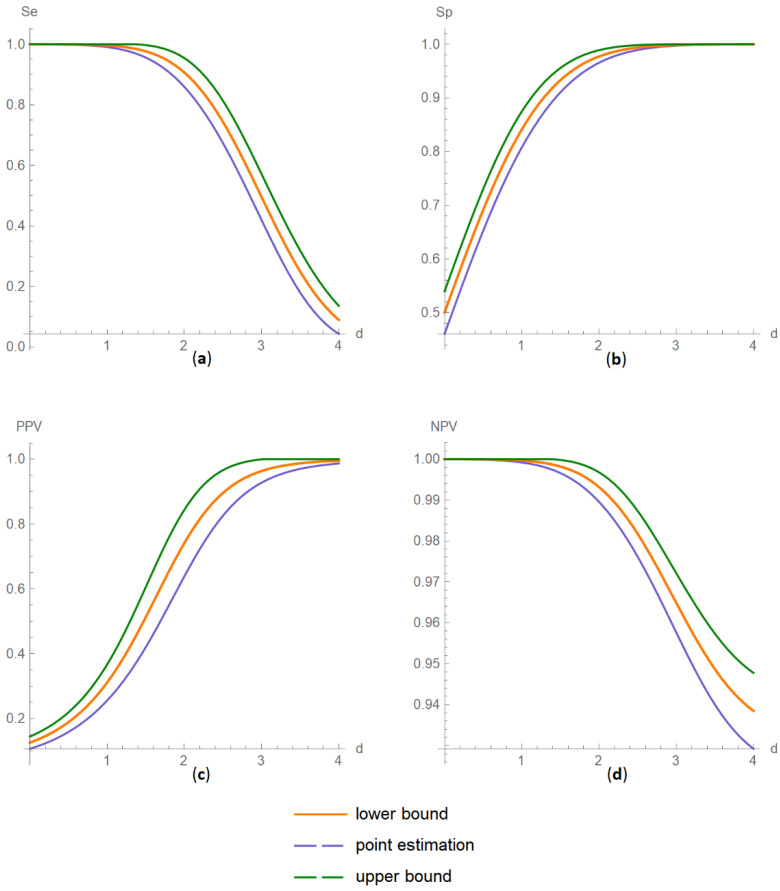
DAM confidence intervals versus diagnostic threshold plots. Plots of confidence intervals of (**a**) sensitivity (*Se*), (**b**) specificity (*Sp*), (**c**) positive predictive value (*PPV*) and (**d**) negative predictive value (*NPV*) versus diagnostic threshold (*d*) curves, with the respective parameter settings in Table 4.

**Figure 18:**
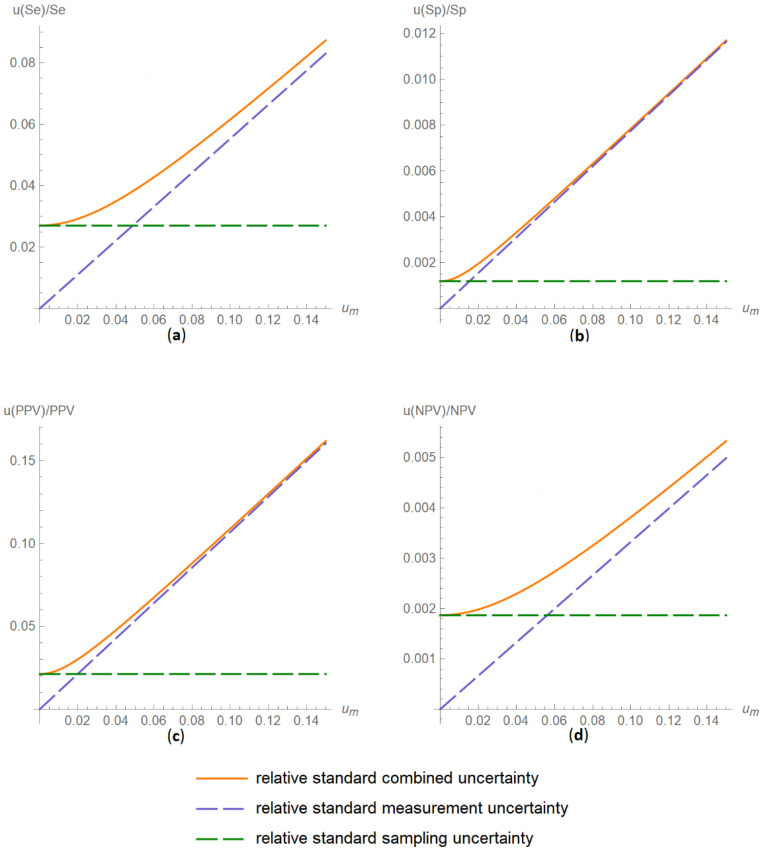
DAM relative standard uncertainties versus measurement uncertainty plots. Plots of relative standard combined, measurement and sampling uncertainties of (**a**) sensitivity (*u*(*Se*)/*Se*), (**b**) specificity (*u*(*Sp*)/*Sp*), (**c**) positive predictive value (*u*(*PPV*)/*PPV*) and (**d**) negative predictive value (*u*(*NPV*)/*NPV*) versus standard measurement uncertainty (*u_m_*) curves, with the respective parameter settings in Table 4.

**Figure 19:**
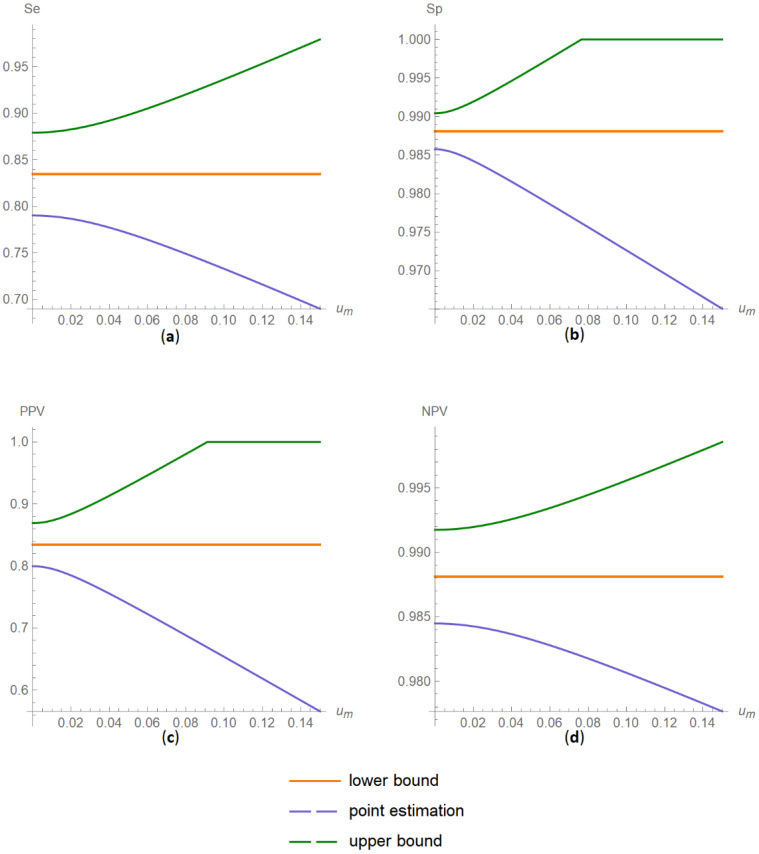
DAM confidence intervals versus measurement uncertainty plots. Plots of confidence intervals of (**a**) sensitivity (*Se*), (**b**) specificity (*Sp*), (**c**) positive predictive value (*PPV*) and (**d**) negative predictive value (*NPV*) versus standard measurement uncertainty (*u_m_*) curves, with the respective parameter settings in Table 4.

**Figure 20:**
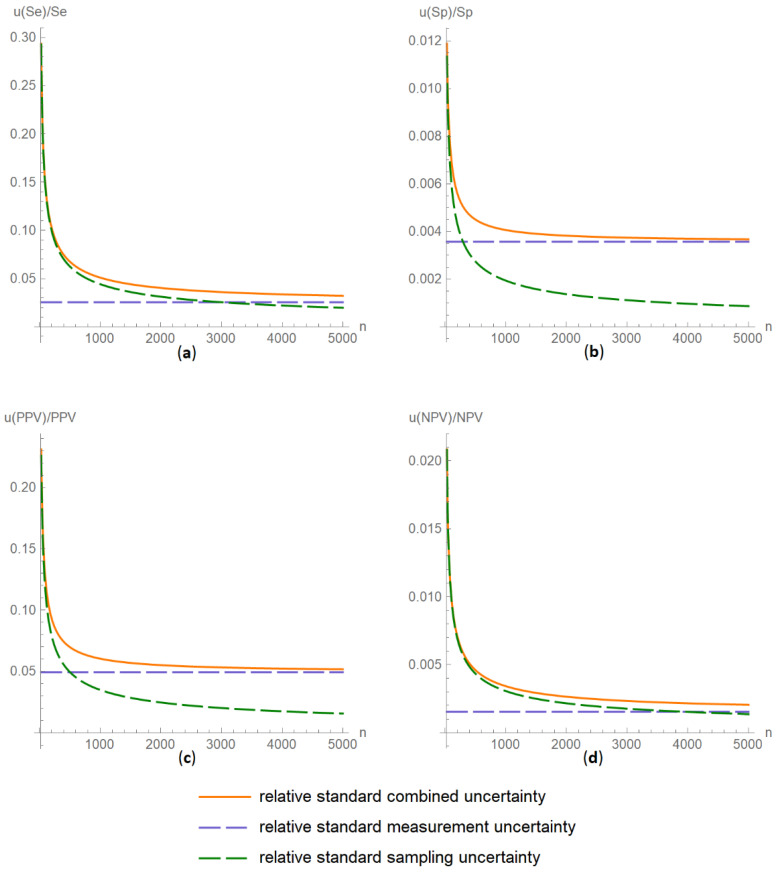
DAM relative standard uncertainties versus population sample size plots. Plots of relative standard combined, measurement and sampling uncertainties of (**a**) sensitivity (*u*(*Se*)/*Se*), (**b**) specificity (*u*(*Sp*)/*Sp*), (**c**) positive predictive value (*u*(*PPV*)/*PPV*) and (**d**) negative predictive value (*u*(*NPV*)/*NPV*) versus total population sample size (*n*) curves, with the respective parameter settings in Table 4.

**Figure 21:**
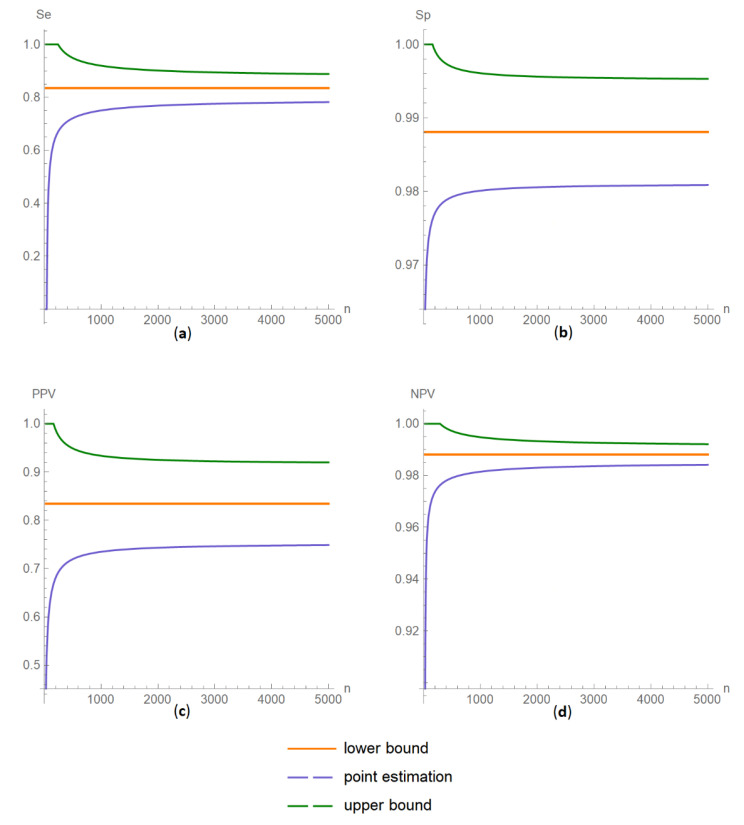
DAM confidence intervals versus population sample size plots. Plots of confidence intervals of (**a**) sensitivity (*Se*), (**b**) specificity (*Sp*), (**c**) positive predictive value (*PPV*) and (**d**) negative predictive value (*NPV*) versus total population sample size (*n*) curves, with the respective parameter settings in Table 4.

**Table 1:** A 2 × 2 contingency table.

		**Populations**	
		**Non-diseased**	**Diseased**
**Test Results**	**Negative**	true negative (*TN*)	false negative (*FN*)
	**Positive**	false positive (*FP*)	true positive (*TP*)

**Table 2:** Natural frequency and probability definitions of diagnostic accuracy measures.

**Measure**	**Natural Frequency Definition**	**Probability Definition**
Sensitivity (*Se*)	$\frac{TP}{FN+TP}$	Pr(T|D)
Specificity (*Sp*)	$\frac{TN}{TN+FP}$	$Pr\left(\bar{T}|\bar{D}\right)$
Positive Predictive Value (*PPV*)	$\frac{TP}{FP+TP}$	Pr(D|T)
Negative Predictive Value (*NPV*)	$\frac{TN}{TN+FN}$	$Pr\left(\bar{D}|\bar{T}\right)$
Overall Diagnostic Accuracy (*ODA*)	$\frac{TN+TP}{TN+FN+TP+FP}$	$\begin{array}{c} Pr\left(D\right)\;Pr\left(T|D\right)\\ +Pr\left(\bar{D}\right)\;\mathrm{Pr}\left(\bar{T}|\bar{D}\right) \end{array}$
Diagnostic Odds Ratio (*DOR*)	$\frac{TN\;TP}{FN\;FP}$	$\frac{\frac{Pr\left(T|D\right)}{Pr\left(\bar{T}|D\right)}}{\frac{Pr\left(T|\bar{D}\right)}{Pr\left(\bar{T}|\bar{D}\right)}}$
Likelihood Ratio for a Positive Result (*LR*+)	$\frac{TP\left(FP+TN\right)}{FP\left(FN+TP\right)}$	$\frac{Pr\left(T|D\right)}{Pr\left(T|\bar{D}\right)}$
Likelihood Ratio for a Negative Result (*LR*−)	$\frac{FN\left(FP+TN\right)}{TN\left(FN+TP\right)}$	$\frac{Pr\left(\bar{T}|D\right)}{Pr\left(\bar{T}|\bar{D}\right)}$
Juden’s Index (*J*)	$\frac{TN\;TP-FN\;FP}{\left(TN+FP\right)\left(FN+TP\right)}$	$Pr\left(T|D\right)+Pr\left(\bar{T}|\bar{D}\right)-1$
Euclidean Distance (*ED*)	$\sqrt{{\left(\frac{FN}{FN+TP}\right)}^{2}+{\left(\frac{FP}{TN+FP}\right)}^{2}}$	$\sqrt{Pr{\left(\bar{T}|D\right)}^{2}+Pr{\left(T|\bar{D}\right)}^{2}}$
Concordance Probability (*CZ*)	$\frac{TN\;TP\;}{\left(TN+FP\right)\left(FN+TP\right)}$	$Pr\left(T|D\right)\;Pr\left(\bar{T}|\bar{D}\right)$

The symbols are explained in Appendix A.

**Table 3:** The parameter settings of Figure 3, Figure 4, Figure 5, Figure 6, Figure 7, Figure 8, Figure 9, Figure 10, Figure 11, Figure 12, Figure 13 and Figure 14.

Settings	Figure 3	Figure 4	Figure 5	Figure 6 and Figure 7	Figure 8	Figure 9 and Figure 10	Figure 11	Figure 12 and Figure 13	Figure 14
p	-	-	0.95	-	0.95	-	0.95	-	0.95
d	1.1–2.5	0–4.0	2.26	2.26	2.26	2.26	2.26	2.26	2.26
r	-	-	-	-	-	0.067	0.067	-	-
$\mu_{D}$	2.99	2.99	2.99	2.99	2.99	2.99	2.99	2.99	2.99
$\sigma_{D}$	0.75	0.75	0.75	0.75	0.75	0.75	0.75	0.75	0.75
$n_{D}$	179	179	179	179	179	-	-	179	179
$\mu_{\bar{D}}$	0.0	0.0	0.0	0.0	0.0	0.0	0.0	0.0	0.0
$\sigma_{\bar{D}}$	1.0	1.0	1.0	1.0	1.0	1.0	1.0	1.0	1.0
$n_{\bar{D}}$	2488	2488	2488	2488	2488	-	-	2488	2488
n	-	-	-	-	-	30–5000	30–5000	-	-
$u_{m}$	0.046	0.046	0.046	0–0.15	0–0.15	0.046	0.046	0.046	0.046
$n_{u}$	-	-	80	-	80	-	80	-	80

The symbols are explained in Appendix A.

**Table 4:** The parameter settings of Figure 15, Figure 16, Figure 17, Figure 18, Figure 19, Figure 20, Figure 21 and Figure 22.

Settings	Figure 15 and Figure 16	Figure 17	Figure 18	Figure 19	Figure 20	Figure 21	Figure 22
p	-	0.95	-	0.95	-	0.95	-
d	0.0–4.0	0.0–4.0	2.26	-	2.26	-	2.26
r	-	-	-	-	0.067	0.067	-
$\mu_{D}$	2.99	2.99	2.99	2.99	2.99	2.99	2.99
$\sigma_{D}$	0.75	0.75	0.75	0.75	0.75	0.75	0.75
$n_{D}$	179	179	179	179	-	-	179
$\mu_{\bar{D}}$	0.0	0.0	0.0	0.0	0.0	0.0	0.0
$\sigma_{\bar{D}}$	1.0	1.0	1.0	1.0	1.0	1.0	1.0
$n_{\bar{D}}$	2488	2488	2488	2488	-	-	2488
*n*	-	-	-	-	30–5000	30–5000	-
$u_{m}$	0.046	0.046	0–0.15	0–0.15	0.046	0.046	0.046
$n_{u}$	-	80	-	80	-	80	-

The symbols are explained in Appendix A.

## Data Availability

The data presented in this study are available in [35].

## Supplementary Materials

The following are available online at https://www.mdpi.com/2075-4418/11/3/406/s1, Software: Diagnostic Uncertainty.nb, Text: Diagnostic Uncertainty Interface.pdf.

## Appendix A

### Notation

1. Populations
  $\bar{D}$ : Non-diseased population
  D : Diseased population
2. Test outcomes
  $\bar{T}$ : Negative test result
  T : Positive test result
  *TN*: True negative test result
  *TP*: True positive test result
  *FN*: False negative test result
  *FP*: False positive test result
3. Parameters
  $m_{P}$ : Mean of the measurand of a test in a sample of population *P*
  $s_{P}$ : Standard deviation of the measurand of a test in a sample of population *P*
  $n_{P}$ : Size of a sample of population *P*
  n : Size of a sample of total population
  $n_{u}$ : Size of a measurements sample
  *r*: Prevalence rate of the disease
  *d*: Diagnostic threshold of a test
  $u_{m\;}$ : Standard measurement uncertainty of a test
  *p*: Confidence level
  *v*: Degrees of freedom
  $v_{eff\;}$ : Effective degrees of freedom
4. Diagnostic accuracy measures: Abbreviations
  *Se*: Sensitivity
  *Sp*: Specificity
  *PPV*: Positive predictive value
  *NPV*: Negative predictive value
  *ODA*: Overall diagnostic accuracy
  *DOR*: Diagnostic odds ratio
  *LR*+: Likelihood ratio for a positive test result
  *LR*−: Likelihood ratio for a negative test result
  *J*: Youden’s index
  *ED*: Euclidean distance of a receiver operating characteristic curve point from the point (0,1)
  *CZ*: Concordance probability
5. Diagnostic accuracy measures: Functions
  se(…) : Sensitivity of a test
  sp(…) : Specificity of a test
  oda(…) : Overall diagnostic accuracy of a test
  ppv(…) : Positive predictive value of a test
  npv(…) : Negative predictive value of a test
  plr(…) : Likelihood ratio for a positive test result
  nlr(…) : Likelihood ratio for a negative test result
  dor(…) : Diagnostic odds ratio of a test
  ed(…) : Euclidean distance of a test
  j(…) : Youden’s index of a test
  cz(…) : Concordance probability of a test
6. Other functions and relations
  *u(x)*: Standard uncertainty of x
  *u_s_*(*x*): Standard sampling uncertainty of x
  *u_m_*(*x*): Standard measurement uncertainty of x
  *u_c_*(*x*): Standard combined uncertainty of x
  *u_i_*(*x*): The *i*^th^ component of the standard combined uncertainty of x
  $u_{c}\left(dam\left(\ldots \right),\ldots \right)$ : Standard combined uncertainty of the diagnostic accuracy measure dam(…)
  Φ(x) : Cumulative distribution function of the standard normal distribution evaluated at x
  Ψ(x,μ,σ) : Cumulative distribution function of a normal distribution with mean μ and standard deviation σ , evaluated at x
  $F_{v}\left(x\right)$ : Cumulative distribution function of the Student’s *t*-distribution with v degrees of freedom, evaluated at x
  erf(x) : Error function, evaluated at x
  erfc(x) : Complementary error function, evaluated at x
  Pr(a) : Probability of an event a
  Pr(a|b) : Probability of an event a given the event b
  $CI_{p}\left(x\right)$ : Confidence interval of x at confidence level p
  $F^{-1}\left(\ldots \right)$ : The inverse function F

## Appendix B

### Appendix B.1. Uncertainty Propagation Rules

$$u\left(x+y\right)=\sqrt{u{\left(x\right)}^{2}+u{\left(y\right)}^{2}}$$

$$u\left(x-y\right)=\sqrt{u{\left(x\right)}^{2}+u{\left(y\right)}^{2}}$$

u(α+x)=u(x)

u(αx)=αu(x)

$$u\left(x^{\alpha }\right)=\left|\alpha \right|x^{\alpha -1}u\left(x\right)$$

$$u\left(x\;y\right)=x\;y\sqrt{{\left(\frac{u\left(x\right)}{x}\right)}^{2}+{\left(\frac{u\left(y\right)}{y}\right)}^{2}}$$

$$u\left(\frac{x}{y}\right)=\frac{x}{y}\sqrt{{\left(\frac{u\left(x\right)}{x}\right)}^{2}+{\left(\frac{u\left(y\right)}{y}\right)}^{2}}$$

$$H\left(x\right)=\int h\left(x\right)dt\Rightarrow u\left(\int_{\alpha }^{\beta }h\left(t\right)dt\right)=u\left(H\left(\beta \right)-H\left(\alpha \right)\right)=\sqrt{{u\left(H\left(\beta \right)\right)}^{2}+u{\left(H\left(\alpha \right)\right)}^{2}}$$

### Appendix B.2. Definitions and Calculations

#### Appendix B.2.1. Error Function

$$erf\left(x\right)=\frac{2}{\sqrt{\pi }}\int_{0}^{x}e^{-t^{2}}dt,\;x\geq 0$$

#### Appendix B.2.2. Complementary Error Function

$$erfc\left(x\right)=1-erf\left(x\right)=\frac{2}{\sqrt{\pi }}\int_{x}^{\infty }e^{-t^{2}}dt,\;x\geq 0$$

#### Appendix B.2.3. Standard Normal Distribution Cumulative Density Function

$$\Phi \left(x\right)=\frac{1}{2\sqrt{\pi }}\int_{-\infty }^{x}e^{-t^{2}}dt=\frac{1}{2}\left(1+erf\left(\frac{x}{\sqrt{2}}\right)\right)=1-\frac{1}{2}\;erfc\left(\frac{x}{\sqrt{2}}\right)$$

#### Appendix B.2.4. Normal Distribution Cumulative Density Function

$$\begin{array}{c} \Psi \left(x,m_{P},s_{P}\right)=\Phi \left(\frac{x-m_{P}}{s_{P}}\right)=\frac{1}{2}\left(1+erf\left(\frac{x-m_{P}}{\sqrt{2}s_{P}}\right)\right)\\ =1-\frac{1}{2}erfc\left(\frac{x-m_{P}}{\sqrt{2}s_{P}}\right) \end{array}$$

#### Appendix B.2.5. Prevalence Rate (*r*)

$$r=\frac{n_{D}}{n_{\bar{D}}+n_{D}}$$

#### Appendix B.2.6. Sensitivity (*Se*)

2.6.1. Measure

$$se\left(d,m_{D},s_{D}\right)=1-\Psi \left(x,m_{D},s_{D}\right)=\;\frac{1}{2}erfc\left(\frac{m_{D}-d}{\sqrt{2}s_{D}}\right)$$

2.6.2. Standard Combined Uncertainty

$$u_{c}\left(se\left(d|m_{D},s_{D}\right)|u,\;n_{D}\right)=\sqrt{\frac{e^{-\frac{{\left(m_{D}-d\right)}^{2}}{s_{D}^{2}}}\left({\left(m_{D}-d\right)}^{2}\left(\frac{s_{D}^{2}}{2\left(n_{D}-1\right)}+u^{2}\right)+s_{D}^{2}\left(\frac{s_{D}^{2}}{n_{D}}+u^{2}\right)\right)}{2\pi \;s_{D}^{4}}}$$

#### Appendix B.2.7. Specificity (*Sp*)

2.7.1. Measure

$$sp\left(d,m_{\bar{D}},s_{\bar{D}}\right)\;=\Psi \left(x,m_{\bar{D}},s_{\bar{D}}\right)=1-\frac{1}{2}erfc\left(\frac{m_{\bar{D}}-d}{\sqrt{2}s_{\bar{D}}}\right)$$

2.7.2. Standard Combined Uncertainty

$$u_{c}\left(sp\left(d,m_{\bar{D}},s_{\bar{D}}\right),u,n_{\bar{D}}\right)\;=\sqrt{\frac{e^{-\frac{{\left(m_{\bar{D}}-d\right)}^{2}}{s_{\bar{D}}^{2}}}\left({\left(m_{\bar{D}}-d\right)}^{2}\left(\frac{s_{\bar{D}}^{2}}{2\left(n_{\bar{D}}-1\right)}+u^{2}\right)+s_{\bar{D}}^{2}\left(\frac{s_{\bar{D}}^{2}}{n_{\bar{D}}}+u^{2}\right)\right)}{2\pi \;s_{\bar{D}}^{4}}}$$

#### Appendix B.2.8. Overall Diagnostic Accuracy (*ODA*)

2.8.1. Measure

$$\begin{array}{c} oda\left(d,m_{D},m_{\bar{D}},s_{D},s_{\bar{D}},n_{D},n_{\bar{D}}\right)\;=\;se\left(v,m_{D},s_{D}\right)\;r+sp\left(v,m_{\bar{D}},s_{\bar{D}}\right)\;\left(1-r\right)\;=\\ \frac{n_{\bar{D}}erfc\left(\frac{m_{\bar{D}}-d}{\sqrt{2}s_{\bar{D}}}\right)+n_{D}erf\left(\frac{m_{D}-d}{\sqrt{2}s_{D}}\right)+n_{D}}{2\left(n_{\bar{D}}+n_{D}\right)} \end{array}$$

2.8.2. Standard Combined Uncertainty

$$u_{c}\left(oda\left(d,m_{D},m_{\bar{D}},s_{D},s_{\bar{D}},n_{D},n_{\bar{D}}\right),u\right)\;=\;\sqrt{\frac{A_{1}+A_{2}+A_{3}+A_{4}+A_{5}}{{\left(n_{\bar{D}}+n_{D}\right)}^{2}}}$$

where:

$$A_{1}=\frac{\left(n_{D}+2\right)\left(n_{\bar{D}}+2\right)\left(n_{\bar{D}}+n_{D}\right){\left(erfc\left(\frac{m_{\bar{D}}-d}{\sqrt{2}s_{\bar{D}}}\right)+erfc\left(\frac{m_{D}-d}{\sqrt{2}s_{D}}\right)-2\right)}^{2}}{{\left(n_{\bar{D}}+n_{D}+4\right)}^{2}}$$

$$A_{2}=\frac{2n_{\bar{D}}e^{-\frac{{\left(m_{\bar{D}}-d\right)}^{2}}{s_{\bar{D}}^{2}}}\left(u^{2}n_{\bar{D}}+s_{\bar{D}}^{2}\right)}{\pi s_{\bar{D}}^{2}}$$

$$A_{3}=\frac{2n_{\bar{D}}^{2}{\left(m_{\bar{D}}-d\right)}^{2}e^{-\frac{{\left(m_{\bar{D}}-t\right)}^{2}}{s_{\bar{D}}^{2}}}\left(\frac{s_{\bar{D}}^{2}}{2\left(n_{\bar{D}}-1\right)}+u^{2}\right)}{\pi s_{\bar{D}}^{4}}$$

$$A_{4}=\frac{2n_{D}e^{-\frac{{\left(m_{D}-d\right)}^{2}}{s_{D}^{2}}}\left(u^{2}n_{D}+s_{D}^{2}\right)}{\pi s_{D}^{2}}$$

$$A_{5}=\frac{2n_{D}^{2}{\left(m_{D}-d\right)}^{2}e^{-\frac{{\left(m_{D}-d\right)}^{2}}{s_{D}^{2}}}\left(\frac{s_{D}^{2}}{2\left(n_{D}-1\right)}+u^{2}\right)}{\pi s_{D}^{4}}$$

#### Appendix B.2.9. Positive Predictive Value (*PPV*)

2.9.1. Measure

$$\begin{array}{cc} dor\left(d,m_{D},m_{\bar{D}},s_{D},s_{\bar{D}}\right) & \;=\;\frac{\frac{se\left(d,m_{D},s_{D}\right)}{1-se\left(d,m_{D},s_{D}\right)}}{\frac{1-sp\left(d,m_{\bar{D}},s_{\bar{D}}\right)}{sp\left(d,m_{\bar{D}},s_{\bar{D}}\right)}}=\\ & \frac{\left(erfc\left(\frac{m_{D}-d}{\sqrt{2}s_{D}}\right)-2\right)erfc\left(\frac{m_{\bar{D}}-d}{\sqrt{2}s_{\bar{D}}}\right)}{erfc\left(\frac{m_{D}-d}{\sqrt{2}s_{D}}\right)\left(erfc\left(\frac{m_{\bar{D}}-d}{\sqrt{2}s_{\bar{D}}}\right)-2\right)} \end{array}$$

2.9.2. Standard Combined Uncertainty

$$u_{c}\left(ppv\left(d,m_{D},m_{\bar{D}},s_{D},s_{\bar{D}},n_{D},n_{\bar{D}}\right),u\right)=\sqrt{\frac{B_{1}+B_{2}+B_{3}+B_{4}+B_{5}}{\pi \;B_{6}}}$$

where:

$$B_{1}=\left(n_{D}-1\right)n_{D}^{2}s_{D}^{4}n_{\bar{D}}^{2}{\left(n_{\bar{D}}+n_{D}+4\right)}^{2}{\left(m_{\bar{D}}-d\right)}^{2}e^{-\frac{{\left(m_{\bar{D}}-d\right)}^{2}}{s_{\bar{D}}^{2}}}\left(2u^{2}\left(n_{\bar{D}}-1\right)+s_{\bar{D}}^{2}\right){\left(erfc\left(\frac{m_{D}-d}{\sqrt{2}s_{D}}\right)-2\right)}^{2}$$

$$B_{2}=2\left(n_{D}-1\right)n_{D}^{2}s_{D}^{4}\left(n_{\bar{D}}-1\right)n_{\bar{D}}{\left(n_{\bar{D}}+n_{D}+4\right)}^{2}s_{\bar{D}}^{2}e^{-\frac{{\left(m_{\bar{D}}-d\right)}^{2}}{s_{\bar{D}}^{2}}}\left(u^{2}n_{\bar{D}}+s_{\bar{D}}^{2}\right){\left(erfc\left(\frac{m_{D}-d}{\sqrt{2}s_{D}}\right)-2\right)}^{2}$$

$$B_{3}=2\left(n_{D}-1\right)n_{D}s_{D}^{2}\left(n_{\bar{D}}-1\right)n_{\bar{D}}^{2}{\left(n_{\bar{D}}+n_{D}+4\right)}^{2}s_{\bar{D}}^{4}e^{-\frac{{\left(m_{D}-d\right)}^{2}}{s_{D}^{2}}}\left(u^{2}n_{D}+s_{D}^{2}\right){\left(erfc\left(\frac{m_{\bar{D}}-d}{\sqrt{2}s_{\bar{D}}}\right)-2\right)}^{2}$$

$$B_{4}=n_{D}^{2}\left(n_{\bar{D}}-1\right)n_{\bar{D}}^{2}{\left(n_{\bar{D}}+n_{D}+4\right)}^{2}s_{\bar{D}}^{4}{\left(m_{D}-d\right)}^{2}e^{-\frac{{\left(m_{D}-d\right)}^{2}}{s_{D}^{2}}}\left(2u^{2}\left(n_{D}-1\right)+s_{D}^{2}\right){\left(erfc\left(\frac{m_{\bar{D}}-d}{\sqrt{2}s_{\bar{D}}}\right)-2\right)}^{2}$$

$$B_{5}=\pi \left(n_{D}-1\right)\left(n_{D}+2\right)s_{D}^{4}\left(n_{\bar{D}}-1\right)\left(n_{\bar{D}}+2\right){\left(n_{\bar{D}}+n_{D}\right)}^{3}s_{\bar{D}}^{4}{\left(erfc\left(\frac{m_{D}-d}{\sqrt{2}s_{D}}\right)-2\right)}^{2}{\left(erfc\left(\frac{m_{\bar{D}}-d}{\sqrt{2}s_{\bar{D}}}\right)-2\right)}^{2}$$

$$B_{6}=\left(n_{D}-1\right)s_{D}^{4}\left(n_{\bar{D}}-1\right){\left(n_{\bar{D}}+n_{D}+4\right)}^{2}s_{\bar{D}}^{4}{\left(n_{\bar{D}}erfc\left(\frac{m_{\bar{D}}-d}{\sqrt{2}s_{\bar{D}}}\right)-2\left(n_{\bar{D}}+n_{D}\right)+n_{D}erfc\left(\frac{m_{D}-d}{\sqrt{2}s_{D}}\right)\right)}^{4}$$

#### Appendix B.2.10. Negative Predictive Value (*NPV*)

2.10.1. Measure

$$\begin{array}{cc} npv\left(d,m_{D},m_{\bar{D}},s_{D},s_{\bar{D}},n_{D},n_{\bar{D}}\right) & \;=\;\frac{sp\left(d,m_{\bar{D}},s_{\bar{D}}\right)\;\left(1-r\right)}{sp\left(d,m_{\bar{D}},s_{\bar{D}}\right)\;\left(1-r\right)+\left(1-se\left(d,m_{D},s_{D}\right)\right)r}\;=\\ & \frac{1}{\frac{n_{D}erfc\left(\frac{m_{D}-d}{\sqrt{2}s_{D}}\right)}{n_{\bar{D}}erfc\left(\frac{m_{\bar{D}}-d}{\sqrt{2}s_{\bar{D}}}\right)}+1} \end{array}$$

2.10.2. Standard Combined Uncertainty

$$u_{c}\left(npv\left(d,m_{D},m_{\bar{D}},s_{D},s_{\bar{D}},n_{D},n_{\bar{D}}\right),u\right)\;=\sqrt{\frac{C_{1}+C_{2}+C_{3}+C_{4}+C_{5}}{{\left(n_{\bar{D}}erfc\left(\frac{m_{\bar{D}}-d}{\sqrt{2}s_{\bar{D}}}\right)+n_{D}erfc\left(\frac{m_{D}-d}{\sqrt{2}s_{D}}\right)\right)}^{4}}}$$

where:

$$C_{1}=\frac{2n_{D}n_{\bar{D}}^{2}e^{-\frac{{\left(m_{D}-d\right)}^{2}}{s_{D}^{2}}}\left(u^{2}n_{D}+s_{D}^{2}\right)erfc{\left(\frac{m_{\bar{D}}-d}{\sqrt{2}s_{\bar{D}}}\right)}^{2}}{\pi s_{D}^{2}}$$

$$C_{2}=\frac{n_{D}^{2}n_{\bar{D}}^{2}{\left(m_{D}-d\right)}^{2}e^{-\frac{{\left(m_{D}-d\right)}^{2}}{s_{D}^{2}}}\left(2u^{2}\left(n_{D}-1\right)+s_{D}^{2}\right)erfc{\left(\frac{m_{\bar{D}}-d}{\sqrt{2}s_{\bar{D}}}\right)}^{2}}{\pi \left(n_{D}-1\right)s_{D}^{4}}$$

$$C_{3}=\frac{2n_{D}^{2}n_{\bar{D}}e^{-\frac{{\left(m_{\bar{D}}-d\right)}^{2}}{s_{\bar{D}}^{2}}}\left(u^{2}n_{\bar{D}}+s_{\bar{D}}^{2}\right)erfc{\left(\frac{m_{D}-d}{\sqrt{2}s_{D}}\right)}^{2}}{\pi s_{\bar{D}}^{2}}$$

$$C_{4}=\frac{n_{D}^{2}n_{\bar{D}}^{2}{\left(m_{\bar{D}}-d\right)}^{2}e^{-\frac{{\left(m_{\bar{D}}-d\right)}^{2}}{s_{\bar{D}}^{2}}}\left(2u^{2}\left(n_{\bar{D}}-1\right)+s_{\bar{D}}^{2}\right)erfc{\left(\frac{m_{D}-d}{\sqrt{2}s_{D}}\right)}^{2}}{\pi \left(n_{\bar{D}}-1\right)s_{\bar{D}}^{4}}$$

$$C_{5}=\frac{\left(n_{D}+2\right)\left(n_{\bar{D}}+2\right){\left(n_{\bar{D}}+n_{D}\right)}^{3}erfc{\left(\frac{m_{D}-d}{\sqrt{2}s_{D}}\right)}^{2}erfc{\left(\frac{m_{\bar{D}}-d}{\sqrt{2}s_{\bar{D}}}\right)}^{2}}{{\left(n_{\bar{D}}+n_{D}+4\right)}^{2}}$$

#### Appendix B.2.11. Diagnostic Odds Ratio (*DOR*)

2.11.1. Measure

$$\begin{array}{cc} dor(d,m_{D},m_{\bar{D}},s_{D},s_{\bar{D}})\; & =\;\frac{\frac{se\left(d,m_{D},s_{D}\right)}{1-se\left(d,m_{D},s_{D}\right)}}{\frac{1-sp\left(d,m_{\bar{D}},s_{\bar{D}}\right)}{sp\left(d,m_{\bar{D}},s_{\bar{D}}\right)}}\\ & =\frac{\left(erfc\left(\frac{m_{D}-d}{\sqrt{2}s_{D}}\right)-2\right)erfc\left(\frac{m_{\bar{D}}-d}{\sqrt{2}s_{\bar{D}}}\right)}{erfc\left(\frac{m_{D}-d}{\sqrt{2}s_{D}}\right)\left(erfc\left(\frac{m_{\bar{D}}-d}{\sqrt{2}s_{\bar{D}}}\right)-2\right)} \end{array}$$

2.11.2. Standard Combined Uncertainty

$$u_{c}\left(dor\left(d,m_{D},m_{\bar{D}},s_{D},s_{\bar{D}}\right),n_{D},n_{\bar{D}},u\right)\;=\;2\sqrt{\frac{D_{1}+D_{2}+D_{3}+D_{4}}{\pi \;D_{5}}}$$

where:

$$D_{1}=2\left(n_{D}-1\right)n_{D}s_{D}^{4}\left(n_{\bar{D}}-1\right)s_{\bar{D}}^{2}e^{-\frac{{\left(m_{\bar{D}}-d\right)}^{2}}{s_{\bar{D}}^{2}}}\left(u^{2}n_{\bar{D}}+s_{\bar{D}}^{2}\right){\left(erfc\left(\frac{m_{D}-d}{\sqrt{2}s_{D}}\right)-2\right)}^{2}erfc{\left(\frac{m_{D}-d}{\sqrt{2}s_{D}}\right)}^{2}$$

$$D_{2}=\left(n_{D}-1\right)n_{D}s_{D}^{4}n_{\bar{D}}{\left(m_{\bar{D}}-d\right)}^{2}e^{-\frac{{\left(m_{\bar{D}}-d\right)}^{2}}{s_{\bar{D}}^{2}}}\left(2u^{2}\left(n_{\bar{D}}-1\right)+s_{\bar{D}}^{2}\right){\left(erfc\left(\frac{m_{D}-d}{\sqrt{2}s_{D}}\right)-2\right)}^{2}erfc{\left(\frac{m_{D}-d}{\sqrt{2}s_{D}}\right)}^{2}$$

$$D_{3}=2\left(n_{D}-1\right)s_{D}^{2}\left(n_{\bar{D}}-1\right)n_{\bar{D}}s_{\bar{D}}^{4}e^{-\frac{{\left(m_{D}-d\right)}^{2}}{s_{D}^{2}}}\left(u^{2}n_{D}+s_{D}^{2}\right){\left(erfc\left(\frac{m_{\bar{D}}-d}{\sqrt{2}s_{\bar{D}}}\right)-2\right)}^{2}erfc{\left(\frac{m_{\bar{D}}-d}{\sqrt{2}s_{\bar{D}}}\right)}^{2}$$

$$D_{4}=n_{D}\left(n_{\bar{D}}-1\right)n_{\bar{D}}s_{\bar{D}}^{4}{\left(m_{D}-d\right)}^{2}e^{-\frac{{\left(m_{D}-d\right)}^{2}}{s_{D}^{2}}}\left(2u^{2}\left(n_{D}-1\right)+s_{D}^{2}\right){\left(erfc\left(\frac{m_{\bar{D}}-d}{\sqrt{2}s_{\bar{D}}}\right)-2\right)}^{2}erfc{\left(\frac{m_{\bar{D}}-d}{\sqrt{2}s_{\bar{D}}}\right)}^{2}$$

$$D_{5}=\left(n_{D}-1\right)n_{D}s_{D}^{4}\left(n_{\bar{D}}-1\right)n_{\bar{D}}s_{\bar{D}}^{4}erfc{\left(\frac{m_{D}-d}{\sqrt{2}s_{D}}\right)}^{4}{\left(erfc\left(\frac{m_{\bar{D}}-d}{\sqrt{2}s_{\bar{D}}}\right)-2\right)}^{4}$$

#### Appendix B.2.12. Likelihood Ratio for a Positive Result (*LR*+)

2.12.1. Measure

$$\begin{array}{c} plr\left(d,m_{D},m_{\bar{D}},s_{D},s_{\bar{D}}\right)\;=\;\frac{se\left(d,m_{D},s_{D}\right)}{1-sp\left(d,m_{\bar{D}},s_{\bar{D}}\right)}\\ =\frac{erf\left(\frac{m_{D}-d}{\sqrt{2}s_{D}}\right)+1}{erf\left(\frac{m_{\bar{D}}-d}{\sqrt{2}s_{\bar{D}}}\right)+1} \end{array}$$

2.12.2. Standard Combined Uncertainty

$$u_{c}\left(plr\left(d,m_{D},m_{\bar{D}},s_{D},s_{\bar{D}}\right),u,n_{D},n_{\bar{D}}\right)\;=\sqrt{\frac{2\left(E_{1}+E_{2}+E_{3}+E_{4}\right)}{\pi \;{\left(erf\left(\frac{m_{\bar{D}}-d}{\sqrt{2}s_{\bar{D}}}\right)+1\right)}^{4}}}$$

where:

$$E_{1}=\frac{e^{-\frac{{\left(m_{D}-d\right)}^{2}}{s_{D}^{2}}}\left(\frac{s_{D}^{2}}{n_{D}}+u^{2}\right){\left(erf\left(\frac{m_{\bar{D}}-d}{\sqrt{2}s_{\bar{D}}}\right)+1\right)}^{2}}{s_{D}^{2}}$$

$$E_{2}=\frac{{\left(m_{D}-d\right)}^{2}e^{-\frac{{\left(m_{D}-d\right)}^{2}}{s_{D}^{2}}}\left(\frac{s_{D}^{2}}{2\left(n_{D}-1\right)}+u^{2}\right){\left(erf\left(\frac{m_{\bar{D}}-d}{\sqrt{2}s_{\bar{D}}}\right)+1\right)}^{2}}{s_{D}^{4}}$$

$$E_{3}=\frac{e^{-\frac{{\left(m_{\bar{D}}-d\right)}^{2}}{s_{\bar{D}}^{2}}}\left(\frac{s_{\bar{D}}^{2}}{n_{\bar{D}}}+u^{2}\right){\left(erf\left(\frac{m_{D}-d}{\sqrt{2}s_{D}}\right)+1\right)}^{2}}{s_{\bar{D}}^{2}}$$

$$E_{4}=\frac{{\left(m_{\bar{D}}-t\right)}^{2}e^{-\frac{{\left(m_{\bar{D}}-d\right)}^{2}}{s_{\bar{D}}^{2}}}\left(\frac{s_{\bar{D}}^{2}}{2\left(n_{\bar{D}}-1\right)}+u^{2}\right){\left(erf\left(\frac{m_{D}-d}{\sqrt{2}s_{D}}\right)+1\right)}^{2}}{s_{\bar{D}}^{4}}$$

#### Appendix B.2.13. Likelihood Ratio for a Negative Result (*LR*−)

2.13.1. Measure

$$\begin{array}{c} nlr\left(d,m_{D},m_{\bar{D}},s_{D},s_{\bar{D}}\right)\;=\;\frac{1-se\left(d,m_{D},s_{D}\right)}{sp\left(d,m_{\bar{D}},s_{\bar{D}}\right)}\\ =\frac{erfc\left(\frac{m_{D}-d}{\sqrt{2}s_{D}}\right)}{erfc\left(\frac{m_{\bar{D}}-d}{\sqrt{2}s_{\bar{D}}}\right)} \end{array}$$

2.13.2. Standard Combined Uncertainty

$$u_{c}\left(nlr\left(d,m_{D},m_{\bar{D}},s_{D},s_{\bar{D}}\right),u,n_{D},n_{\bar{D}}\right)\;=\sqrt{\frac{2\left(F_{1}+F_{2}+F_{3}+F_{4}\right)}{\pi \;erfc{\left(\frac{m_{\bar{D}}-d}{\sqrt{2}s_{\bar{D}}}\right)}^{4}}}$$

where:

$$F_{1}=\frac{e^{-\frac{{\left(m_{D}-d\right)}^{2}}{s_{D}^{2}}}\left(\frac{s_{D}^{2}}{n_{D}}+u^{2}\right)erfc{\left(\frac{m_{\bar{D}}-d}{\sqrt{2}s_{\bar{D}}}\right)}^{2}}{s_{D}^{2}}$$

$$F_{2}=\frac{{\left(m_{D}-d\right)}^{2}e^{-\frac{{\left(m_{D}-d\right)}^{2}}{s_{D}^{2}}}\left(\frac{s_{D}^{2}}{2\left(n_{D}-1\right)}+u^{2}\right)erfc{\left(\frac{m_{\bar{D}}-d}{\sqrt{2}s_{\bar{D}}}\right)}^{2}}{s_{D}^{4}}$$

$$F_{3}=\frac{e^{-\frac{{\left(m_{\bar{D}}-d\right)}^{2}}{s_{\bar{D}}^{2}}}\left(\frac{s_{\bar{D}}^{2}}{n_{\bar{D}}}+u^{2}\right)erfc{\left(\frac{m_{D}-d}{\sqrt{2}s_{D}}\right)}^{2}}{s_{\bar{D}}^{2}}$$

$$F_{4}=\frac{{\left(m_{\bar{D}}-d\right)}^{2}e^{-\frac{{\left(m_{\bar{D}}-d\right)}^{2}}{s_{\bar{D}}^{2}}}\left(\frac{s_{\bar{D}}^{2}}{2\left(n_{\bar{D}}-1\right)}+u^{2}\right)erfc{\left(\frac{m_{D}-d}{\sqrt{2}s_{D}}\right)}^{2}}{s_{\bar{D}}^{4}}$$

#### Appendix B.2.14. Yuden’s Index (*J*)

2.14.1. Measure

$$\begin{array}{c} j\left(d,m_{D},m_{\bar{D}},s_{D},s_{\bar{D}}\right)\;=\;se\left(d,m_{D},s_{D}\right)+sp\left(d,m_{\bar{D}},s_{\bar{D}}\right)-1\\ =\frac{1}{2}\left(erf\left(\frac{m_{D}-d}{\sqrt{2}s_{D}}\right)-erf\left(\frac{m_{\bar{D}}-d}{\sqrt{2}s_{\bar{D}}}\right)\right) \end{array}$$

2.14.2. Standard Combined Uncertainty

$$u_{c}\left(j\left(d,m_{D},m_{\bar{D}},s_{D},s_{\bar{D}}\right),u,n_{D},n_{\bar{D}}\right)=\;\frac{\sqrt{G_{1}+G_{2}+G_{3}+G_{4}}}{\sqrt{2\pi }}$$

where:

$$G_{1}=\frac{e^{-\frac{{\left(m_{\bar{D}}-d\right)}^{2}}{s_{\bar{D}}^{2}}}\left(\frac{s_{\bar{D}}^{2}}{n_{\bar{D}}}+u^{2}\right)}{s_{\bar{D}}^{2}}$$

$$G_{2}=\frac{{\left(m_{\bar{D}}-d\right)}^{2}e^{-\frac{{\left(m_{\bar{D}}-d\right)}^{2}}{s_{\bar{D}}^{2}}}\left(\frac{s_{\bar{D}}^{2}}{2\left(n_{\bar{D}}-1\right)}+u^{2}\right)}{s_{\bar{D}}^{4}}$$

$$G_{3}=\frac{e^{-\frac{{\left(m_{D}-d\right)}^{2}}{s_{D}^{2}}}\left(\frac{s_{D}^{2}}{n_{D}}+u^{2}\right)}{s_{D}^{2}}$$

$$G_{4}=\frac{{\left(m_{D}-d\right)}^{2}e^{-\frac{{\left(m_{D}-d\right)}^{2}}{s_{D}^{2}}}\left(\frac{s_{D}^{2}}{2\left(n_{D}-1\right)}+u^{2}\right)}{s_{D}^{4}}$$

#### Appendix B.2.15. Euclidean Distance (*ED*)

2.15.1. Measure

$$\begin{array}{c} mced\left(d,m_{D},m_{\bar{D}},s_{D},s_{\bar{D}}\right)\;=\;\sqrt{{\left(1-se\left(d,m_{D},s_{D}\right)\right)}^{2}+{\left(1-sp\left(d,m_{\bar{D}},s_{\bar{D}}\right)\right)}^{2}}\\ =\frac{1}{2}\sqrt{{\left(erf\left(\frac{m_{\bar{D}}-d}{\sqrt{2}s_{\bar{D}}}\right)+1\right)}^{2}+erfc{\left(\frac{m_{D}-d}{\sqrt{2}s_{D}}\right)}^{2}} \end{array}$$

2.15.2. Standard Combined Uncertainty

$$u_{c}\left(ed\left(d,m_{D},m_{\bar{D}},s_{D},s_{\bar{D}}\right),u,n_{D},n_{\bar{D}}\right)\;=\;\sqrt{\frac{H_{1}+H_{2}+H_{3}+H_{4}}{2\pi \;H_{5}}}$$

where:

$$H_{1}=\frac{e^{-\frac{{\left(m_{\bar{D}}-d\right)}^{2}}{s_{\bar{D}}^{2}}}\left(\frac{s_{\bar{D}}^{2}}{n_{\bar{D}}}+u^{2}\right){\left(erf\left(\frac{m_{\bar{D}}-d}{\sqrt{2}s_{\bar{D}}}\right)+1\right)}^{2}}{s_{\bar{D}}^{2}}$$

$$H_{2}=\frac{{\left(m_{\bar{D}}-d\right)}^{2}e^{-\frac{{\left(m_{\bar{D}}-d\right)}^{2}}{s_{\bar{D}}^{2}}}\left(\frac{s_{\bar{D}}^{2}}{2\left(n_{\bar{D}}-1\right)}+u^{2}\right){\left(erf\left(\frac{m_{\bar{D}}-d}{\sqrt{2}s_{\bar{D}}}\right)+1\right)}^{2}}{s_{\bar{D}}^{4}}$$

$$H_{3}=\frac{e^{-\frac{{\left(m_{D}-d\right)}^{2}}{s_{D}^{2}}}\left(\frac{s_{D}^{2}}{n_{D}}+u^{2}\right)erfc{\left(\frac{m_{D}-d}{\sqrt{2}s_{D}}\right)}^{2}}{s_{D}^{2}}$$

$$H_{4}=\frac{{\left(m_{D}-d\right)}^{2}e^{-\frac{{\left(m_{D}-d\right)}^{2}}{s_{D}^{2}}}\left(\frac{s_{D}^{2}}{2\left(n_{D}-1\right)}+u^{2}\right)erfc{\left(\frac{m_{D}-d}{\sqrt{2}s_{D}}\right)}^{2}}{s_{D}^{4}}$$

$$H_{5}={\left(erf\left(\frac{m_{\bar{D}}-d}{\sqrt{2}s_{\bar{D}}}\right)+1\right)}^{2}+erfc{\left(\frac{m_{D}-d}{\sqrt{2}s_{D}}\right)}^{2}$$

#### Appendix B.2.16. Concordance Probability (*CZ*)

2.16.1. Measure

$$\begin{array}{c} cz\left(d,m_{D},m_{\bar{D}},s_{D},s_{\bar{D}}\right)\;=\;se\left(d,m_{D},s_{D}\right)\;sp\left(d,m_{\bar{D}},s_{\bar{D}}\right)=\\ -\frac{1}{4}\left(erfc\left(\frac{m_{D}-d}{\sqrt{2}s_{D}}\right)-2\right)erfc\left(\frac{m_{\bar{D}}-d}{\sqrt{2}s_{\bar{D}}}\right) \end{array}$$

2.16.2. Standard Combined Uncertainty

$$u_{c}\left(cz\left(d,m_{D},m_{\bar{D}},s_{D},s_{\bar{D}}\right),u,n_{D},n_{\bar{D}}\right)=\frac{1}{2}\sqrt{\frac{I_{1}+I_{2}+I_{3}+I_{4}}{2\pi }}$$

where:

$$I_{1}=\frac{e^{-\frac{{\left(m_{D}-d\right)}^{2}}{s_{D}^{2}}}\left(\frac{s_{D}^{2}}{n_{D}}+u^{2}\right)erfc{\left(\frac{m_{\bar{D}}-d}{\sqrt{2}s_{\bar{D}}}\right)}^{2}}{s_{D}^{2}}$$

$$I_{2}=\frac{{\left(m_{D}-d\right)}^{2}e^{-\frac{{\left(m_{D}-d\right)}^{2}}{s_{D}^{2}}}\left(\frac{s_{D}^{2}}{2\left(n_{D}-1\right)}+u^{2}\right)erfc{\left(\frac{m_{\bar{D}}-d}{\sqrt{2}s_{\bar{D}}}\right)}^{2}}{s_{D}^{4}}$$

$$I_{3}=\frac{e^{-\frac{{\left(m_{\bar{D}}-d\right)}^{2}}{s_{\bar{D}}^{2}}}\left(\frac{s_{\bar{D}}^{2}}{n_{\bar{D}}}+u^{2}\right){\left(erfc\left(\frac{m_{D}-d}{\sqrt{2}s_{D}}\right)-2\right)}^{2}}{s_{\bar{D}}^{2}}$$

$$I_{4}=\frac{{\left(m_{\bar{D}}-v\right)}^{2}e^{-\frac{{\left(m_{\bar{D}}-d\right)}^{2}}{s_{\bar{D}}^{2}}}\left(\frac{s_{\bar{D}}^{2}}{2\left(n_{\bar{D}}-1\right)}+u^{2}\right){\left(erfc\left(\frac{m_{D}-d}{\sqrt{2}s_{D}}\right)-2\right)}^{2}}{s_{\bar{D}}^{4}}$$

## Appendix C

### Software Availability and Requirements

Program name: *Diagnostic Uncertainty*
Project home page: https://www.hcsl.com/Tools/Uncertainty/ (accessed 24 February 2021)
Operating systems: Microsoft Windows, Linux, Apple iOS
Programming language: Wolfram Language
Other software requirements: Wolfram Player^®^, freely available at: https://www.wolfram.com/player/ (accessed 12 February 2021) or Wolfram Mathematica^®^
System requirements: Intel^®^ i7™ or equivalent CPU and 16 GB of RAM
License: Attribution—Noncommercial—ShareAlike 4.0 International Creative Commons License
