# Correction: Chatzimichail, T.; Hatjimihail, A.T. A Software Tool for Calculating the Uncertainty of Diagnostic Accuracy Measures. *Diagnostics* 2021, *11*, 406

**DOI:** 10.3390/diagnostics14050467

**Published:** 2024-02-21

**Authors:** Theodora Chatzimichail, Aristides T. Hatjimihail

**Affiliations:** Hellenic Complex Systems Laboratory, Kostis Palamas 21, 66131 Drama, Greece; tc@hcsl.com

## Figure/Table Legend

In the original publication [1], there was a mistake in the legend for Figure 13. The term submodule was misprinted, and the term relative was omitted from the first sentence of the legend. The correct legend appears below.

**Figure 13.** DAM relative uncertainty calculator submodule screenshot. Calculated relative standard combined, and the measurement and sampling uncertainty of diagnostic accuracy measures, with the settings shown on the left. The respective parameter settings are also shown in Table 3.

## Text Correction

There was an error in the original publication regarding an equation in an uncertainty propagation rule. This error has been corrected in the original publication [1], specifically in the last line. The corrected equation is as follows:

$$H\left(x\right)=\int h\left(x\right)dt\Rightarrow u\left(\int_{\alpha }^{\beta }h\left(t\right)dt\right)=u\left(H\left(\beta \right)-H\left(\alpha \right)\right)=\sqrt{{u\left(H\left(\beta \right)\right)}^{2}+u{\left(H\left(\alpha \right)\right)}^{2}}$$

The authors state that the results and the scientific conclusions are unaffected. This correction was approved by the Academic Editor. The original publication has also been updated.
