# Supplementary material for: A Software Tool for Calculating the Uncertainty of Diagnostic Accuracy Measures
Source: Diagnostics (Basel). 2021 Feb 27;11(3):406. doi: 10.3390/diagnostics11030406 (PMC7997227; doi:10.3390/diagnostics11030406)
Supplement: Supplementary file 1 [file diagnostics-11-00406-s001.zip › Diagnostic Uncertainty Interface.pdf]

Diagnostic Uncertainty

A software tool for calculating the uncertainty of diagnostic accuracy measures

Version 1.0.2

Theodora Chatzimichail, MD [a], *Aristides T. Hatjimihail, MD, PhD* [b]

Hellenic Complex Systems Laboratory, Kostis Palamas 21, Drama 66131, Greece, [a]tc@hcsl.com, [b]ath@hcsl.com

1 INTERFACE OF THE PROGRAM *DIAGNOSTIC UNCERTAINTY*

1.1 ABOUT THE PROGRAM CONTROLS

The numerical settings are defined by the user with menus or sliders. Sliders can be finely manipulated by holding down the alt key or opt key while dragging the mouse. They be even more finely manipulated by also holding the *shift* and/or *ctrl* keys.

Dragging with the mouse rotates the three-dimensional plots, while dragging with the mouse while pressing the *ctrl*, *alt*, or *opt* keys zooms in or out.

1.2 RANGE OF PARAMETERS

1.2.1 Input parameters

p: 0.90 – 0.999

d: $\max\{\mu_D - 4,\ \mu_{\overline{D}} - 4\sigma_{\overline{D}}\} - \min\{\mu_D + 4\sigma_D,\ \mu_{\overline{D}} + 4\sigma_{\overline{D}}\}$

$\mu_{\overline{D}}$: 0 – 6

$\sigma_{\overline{D}}$: 1 – 6

$n_{\overline{D}}$: 2 – 10000

μ_D: 0.1 – 6

σ_D: 0.1 – 6

n_D : 2 – 10000

r: 0.001 – 0.999

u_m : 0 – 6

n_u : 20 – 1000

1.2.2 Range of the coordinates of the plots

There are two options for the range of coordinates to be included in each plot:

1) Full: All the calculated coordinate points are plotted.
2) Partial: The distribution of coordinate values is found and any points sufficiently far out in the distribution are not considered.

1.2.3 Abscissas

1.2.3.1 Full range

d: $(\max\{ \mu_D - 4\sigma_{\overline{D}},\ \mu_{\overline{D}} - 4\sigma_{\overline{D}} \} - \min\{ \mu_D + 4\sigma_D,\ \mu_{\overline{D}} + 4\sigma_{\overline{D}} \})$

u_m : (0 – 0.5 $\sigma_{\overline{D}}$)

n : (max $\{\llbracket \frac{2}{r} \rrbracket, \llbracket \frac{2}{1-r} \rrbracket$, 20} – max {10 $\llbracket \frac{2}{r} \rrbracket$,10 $\llbracket \frac{2}{1-r} \rrbracket$, 10000})

1.2.3.2 Partial range

d: $(\max\{ \mu_D - 2.5\sigma_{\overline{D}},\ \mu_{\overline{D}} - 2.5\sigma_{\overline{D}} \} - \min\{ \mu_D + 2.5\sigma_D,\ \mu_{\overline{D}} + 2.5\sigma_{\overline{D}} \})$

u_m : (0 – 0.15 $\sigma_{\overline{D}}$)

n : (max $\{\llbracket \frac{2}{r} \rrbracket, \llbracket \frac{2}{1-r} \rrbracket$, 20} – max {5 $\llbracket \frac{2}{r} \rrbracket$, 5 $\llbracket \frac{2}{1-r} \rrbracket$, 5000})

1.3 INPUT AND OUTPUT

The program provides in six modules and nine modules plots and tables of the uncertainty and the resultant confidence intervals of diagnostic accuracy measures of a screening or diagnostic test for a measurand, applied at a single point in time in samples of diseased and a non-diseased population.

Singularity points are excluded from the plots.

Indeterminate results of the calculation modules represent numerical quantities whose magnitudes cannot be determined, because they are either too small or too large.

1.3.1 Plots

1.3.1.1 Plots vs diagnostic threshold module

1.3.1.1.1 Diagnostic accuracy measures uncertainty plots

1.3.1.1.1.1 Input

The user defines:

1) The diseased and non-diseased population samples parameters:
   a) The measurand means,
   b) The measurand standard deviations,
   c) The sizes of the samples.
2) The diagnostic accuracy measure:
   a) Sensitivity (*Se*),
   b) Specificity (*Sp*),
   c) Overall diagnostic accuracy (*ODA*),
   d) Positive predictive value (*PPV*),
   e) Negative predictive value (*NPV*),
   f) Diagnostic odds ratio (*DOR*),
   g) Likelihood ratio for a positive test result (*LR* +),
   h) Likelihood ratio for a negative test result (*LR* -),
   i) Youden’s index (*J*),
   j) Euclidean distance (*ED*),
   k) Concordance probability (*CZ*).
3) The standard measurement uncertainty of the test.

4) The range of the coordinate points to be plotted:
    a) Full,
    b) Partial.

1.3.1.1.1.2 Output

Plots of the values of the:

1) Standard combined uncertainty,
2) Standard measurement uncertainty,
3) Standard sampling uncertainty

of the measure versus diagnostic threshold (*d*).

1.3.1.1.2 Diagnostic accuracy measures relative uncertainty plots

1.3.1.1.2.1 Input

The user defines:

1) The diseased and non-diseased population samples parameters:
    a) The measurand means,
    b) The measurand standard deviations,
    c) The sizes of the samples.
2) The diagnostic accuracy measure:
    a) Sensitivity (*Se*),
    b) Specificity (*Sp*),
    c) Overall diagnostic accuracy (*ODA*),
    d) Positive predictive value (*PPV*),
    e) Negative predictive value (*NPV*),
    f) Diagnostic odds ratio (*DOR*),
    g) Likelihood ratio for a positive test result (*LR* +),
    h) Likelihood ratio for a negative test result (*LR* -),
    i) Youden's index (*J*),
    j) Euclidean distance (*ED*),
    k) Concordance probability (*CZ*).
3) The standard measurement uncertainty of the test.
4) The range of the coordinate points to be plotted:
    a) Full,
    b) Partial.

1.3.1.1.2.2 Output

Plots of the values of the:

1) Relative standard combined uncertainty,
2) Relative standard measurement uncertainty,
3) Relative standard sampling uncertainty

of the measure versus diagnostic threshold (*d*).

1.3.1.1.3 Diagnostic accuracy measures confidence intervals plots

1.3.1.1.3.1 Input

The user defines:

1) The confidence level (*p*).
2) The diseased and non-diseased population samples parameters:
    a) The measurand means,

   b) The measurand standard deviations,
   c) The sizes of the samples.
3) The diagnostic accuracy measure:
   a) Sensitivity (*Se*),
   b) Specificity (*Sp*),
   c) Overall diagnostic accuracy (*ODA*),
   d) Positive predictive value (*PPV*),
   e) Negative predictive value (*NPV*),
   f) Diagnostic odds ratio (*DOR*),
   g) Likelihood ratio for a positive test result (*LR* +),
   h) Likelihood ratio for a negative test result (*LR* -),
   i) Youden's index (*J*),
   j) Euclidean distance (*ED*),
   k) Concordance probability (*CZ*).
4) The standard measurement uncertainty of the test.
5) The size of the sample of the measurements for the estimation of the measurement uncertainty.
6) The range of the coordinate points to be plotted:
   a) Full,
   b) Partial.

1.3.1.1.3.2 Output

Plots of the values of the:

1) Lower bound,
2) Point estimation,
3) Upper bound

of the measure versus diagnostic threshold (*d*), at the selected confidence level.

1.3.1.2 Plots vs measurement uncertainty module

1.3.1.2.1 Diagnostic accuracy measures uncertainty plots module

1.3.1.2.1.1 Input

The user defines:

1) The diseased and non-diseased population samples parameters:
   a) The measurand means,
   b) The measurand standard deviations,
   c) The sizes of the samples.
2) The diagnostic accuracy measure:
   a) Sensitivity (*Se*),
   b) Specificity (*Sp*),
   c) Overall diagnostic accuracy (*ODA*),
   d) Positive predictive value (*PPV*),
   e) Negative predictive value (*NPV*),
   f) Diagnostic odds ratio (*DOR*),
   g) Likelihood ratio for a positive test result (*LR* +),
   h) Likelihood ratio for a negative test result (*LR* -),
   i) Youden's index (*J*),
   j) Euclidean distance (*ED*),
   k) Concordance probability (*CZ*).
3) The range of the coordinate points to be plotted:
   a) Full,
   b) Partial.

1.3.1.2.1.2 *Output*

Plots of the values of the:

1) Standard combined uncertainty,
2) Standard measurement uncertainty,
3) Standard sampling uncertainty

of the measure versus standard measurement uncertainty (u_m).

1.3.1.2.2 Diagnostic accuracy measures relative uncertainty plots

1.3.1.2.2.1 *Input*

The user defines:

1) The diseased and non-diseased population samples parameters:
    a) The prevalence rate of the disease condition,
    b) The measurand means,
    c) The measurand standard deviations.
2) The diagnostic accuracy measure:
    a) Sensitivity (*Se*),
    b) Specificity (*Sp*),
    c) Overall diagnostic accuracy (*ODA*),
    d) Positive predictive value (*PPV*),
    e) Negative predictive value (*NPV*),
    f) Diagnostic odds ratio (*DOR*),
    g) Likelihood ratio for a positive test result (*LR* +),
    h) Likelihood ratio for a negative test result (*LR* -),
    i) Youden's index (*J*),
    j) Euclidean distance (*ED*),
    k) Concordance probability (*CZ*).
3) The standard measurement uncertainty of the test.
4) The range of the coordinate points to be plotted:
    a) Full,
    b) Partial.

1.3.1.2.2.2 *Output*

Plots of the values of the:

1) Relative standard combined uncertainty,
2) Relative standard measurement uncertainty,
3) Relative standard sampling uncertainty

of the measure versus standard measurement uncertainty (u_m).

1.3.1.2.3 Diagnostic accuracy measures confidence intervals plots module

1.3.1.2.3.1 *Input*

The user defines:

1) The confidence level (*p*).
2) The diseased and non-diseased population samples parameters:
    a) The measurand means,
    b) The measurand standard deviations,
    c) The sizes of the samples.
3) The diagnostic accuracy measure:
    a) Sensitivity (*Se*),

   b) Specificity (*Sp*),
   c) Overall diagnostic accuracy (*ODA*),
   d) Positive predictive value (*PPV*),
   e) Negative predictive value (*NPV*),
   f) Diagnostic odds ratio (*DOR*),
   g) Likelihood ratio for a positive test result (*LR* +),
   h) Likelihood ratio for a negative test result (*LR* -),
   i) Youden's index (*J*),
   j) Euclidean distance (*ED*),
   k) Concordance probability (*CZ*).
4) The size of the sample of the measurements for the estimation of the measurement uncertainty.
5) The range of the coordinate points to be plotted:
   a) Full,
   b) Partial.

1.3.1.2.3.2 Output

Plots of the values of the:

1) Lower bound,
2) Point estimation,
3) Upper bound

of the measure versus standard measurement uncertainty (u_m), at the selected confidence level.

1.3.1.3 Plots vs population size module

1.3.1.3.1 Diagnostic accuracy measures uncertainty plots

1.3.1.3.1.1 Input

The user defines:

4) The diseased and non-diseased population samples parameters:
   a) The prevalence rate of the disease condition,
   b) The measurand means,
   c) The measurand standard deviations.
5) The diagnostic accuracy measure:
   a) Sensitivity (*Se*),
   b) Specificity (*Sp*),
   c) Overall diagnostic accuracy (*ODA*),
   d) Positive predictive value (*PPV*),
   e) Negative predictive value (*NPV*),
   f) Diagnostic odds ratio (*DOR*),
   g) Likelihood ratio for a positive test result (*LR* +),
   h) Likelihood ratio for a negative test result (*LR* -),
   i) Youden's index (*J*),
   j) Euclidean distance (*ED*),
   k) Concordance probability (*CZ*).
6) The standard measurement uncertainty of the test.
7) The range of the coordinate points to be plotted:
   a) Full,
   b) Partial.

1.3.1.3.1.2 Output

Plots of the values of the:

1) Standard combined uncertainty,

2) Standard measurement uncertainty,
3) Standard sampling uncertainty

of the measure versus total population size (*n*).

1.3.1.3.2 Diagnostic accuracy measures relative uncertainty plots module

1.3.1.3.2.1 Input

The user defines:

1) The diseased and non-diseased population samples parameters:
    a) The prevalence rate of the disease condition,
    b) The measurand means,
    c) The measurand standard deviations.
2) The diagnostic accuracy measure:
    a) Sensitivity (*Se*),
    b) Specificity (*Sp*),
    c) Overall diagnostic accuracy (*ODA*),
    d) Positive predictive value (*PPV*),
    e) Negative predictive value (*NPV*),
    f) Diagnostic odds ratio (*DOR*),
    g) Likelihood ratio for a positive test result (*LR* +),
    h) Likelihood ratio for a negative test result (*LR* -),
    i) Youden's index (*J*),
    j) Euclidean distance (*ED*),
    k) Concordance probability (*CZ*).
3) The standard measurement uncertainty of the test.
4) The range of the coordinate points to be plotted:
    a) Full,
    b) Partial.

1.3.1.3.2.2 Output

Plots of the values of the:

1) Relative standard combined uncertainty,
2) Relative standard measurement uncertainty,
3) Relative standard sampling uncertainty

of the measure versus total population size (*n*).

1.3.1.3.3 Diagnostic accuracy measures confidence intervals plots

1.3.1.3.3.1 Input

The user defines:

1) The confidence level (*p*).
2) The diseased and non-diseased population samples parameters:
    a) The prevalence rate of the disease condition,
    b) The measurand means,
    c) The measurand standard deviations.
3) The diagnostic accuracy measure:
    a) Sensitivity (*Se*),
    b) Specificity (*Sp*),
    c) Overall diagnostic accuracy (*ODA*),
    d) Positive predictive value (*PPV*),
    e) Negative predictive value (*NPV*),

f) Diagnostic odds ratio (*DOR*),
g) Likelihood ratio for a positive test result (*LR* +),
h) Likelihood ratio for a negative test result (*LR* -),
i) Youden's index (*J*),
j) Euclidean distance (*ED*),
k) Concordance probability (*CZ*).
4) The standard measurement uncertainty of the test.
5) The size of the sample of the measurements for the estimation of the measurement uncertainty.
6) The range of the coordinate points to be plotted:
   a) Full,
   b) Partial.

1.3.1.3.3.2 Output

Plots of the values of the:

1) Lower bound,
2) Point estimation,
3) Upper bound

of the measure versus total population size (*n*) at the selected confidence level.

1.3.2 Calculators

1.3.2.1 Diagnostic accuracy measures uncertainty calculator module

1.3.2.1.1 Input

1) The diagnostic threshold (*d*).
2) The diseased and non-diseased population samples parameters:
   a) The measurand means,
   b) The measurand standard deviations,
   c) The sizes of the samples.
3) The standard measurement uncertainty of the test.

1.3.2.1.2 Output

A table of the values of:

1) The standard combined uncertainty,
2) The standard measurement uncertainty,
3) The standard sampling uncertainty

of the following diagnostic accuracy measures at the selected diagnostic threshold:

1) Sensitivity (*Se*),
2) Specificity (*Sp*),
3) Overall diagnostic accuracy (*ODA*),
4) Positive predictive value (*PPV*),
5) Negative predictive value (*NPV*),
6) Diagnostic odds ratio (*DOR*),
7) Likelihood ratio for a positive test result (*LR* +),
8) Likelihood ratio for a negative test result (*LR* -),
9) Youden's index (*J*),
10) Euclidean distance (*ED*),
11) Concordance probability (*CZ*).

1.3.2.2 Diagnostic accuracy measures relative uncertainty calculator module

1.3.2.2.1 Input

1) The diagnostic threshold (*d*).
2) The diseased and non-diseased population samples parameters:
   a) The measurand means,
   b) The measurand standard deviations,
   c) The sizes of the samples,
3) The standard measurement uncertainty of the test.

1.3.2.2.2 Output

A table of the values of:

1) The relative standard combined uncertainty,
2) The relative standard measurement uncertainty,
3) The relative standard sampling uncertainty

of the following diagnostic accuracy measures at the selected diagnostic threshold:

1) Sensitivity (*Se*),
2) Specificity (*Sp*),
3) Overall diagnostic accuracy (*ODA*),
4) Positive predictive value (*PPV*),
5) Negative predictive value (*NPV*),
6) Diagnostic odds ratio (*DOR*),
7) Likelihood ratio for a positive test result (*LR* +),
8) Likelihood ratio for a negative test result (*LR* -),
9) Youden's index (*J*),
10) Euclidean distance (*ED*),
11) Concordance probability (*CZ*).

1.3.2.3 Diagnostic accuracy measures confidence intervals calculator module

1.3.2.3.1 Input

1) The confidence level (*p*).
2) The diagnostic threshold (*d*).
3) The diseased and non-diseased population samples parameters:
   a) The measurand means,
   b) The measurand standard deviations.
   c) The sizes of the samples.
4) The standard measurement uncertainty of the test.
5) The size of the sample of the measurements for the estimation of the measurement uncertainty.

1.3.2.3.2 Output

A table of the values and the confidence intervals of the following diagnostic accuracy measures at the selected confidence level and diagnostic threshold:

1) Sensitivity (*Se*),
2) Specificity (*Sp*),
3) Overall diagnostic accuracy (*ODA*),
4) Positive predictive value (*PPV*),
5) Negative predictive value (*NPV*),
6) Diagnostic odds ratio (*DOR*),
7) Likelihood ratio for a positive test result (*LR* +),
8) Likelihood ratio for a negative test result (*LR* -),

9) Youden's index (J),
10) Euclidean distance (ED),
11) Concordance probability (CZ).
